# A determination of the charm content of the proton: The NNPDF Collaboration

**DOI:** 10.1140/epjc/s10052-016-4469-y

**Published:** 2016-11-24

**Authors:** Richard D. Ball, Valerio Bertone, Marco Bonvini, Stefano Carrazza, Stefano Forte, Alberto Guffanti, Nathan P. Hartland, Juan Rojo, Luca Rottoli

**Affiliations:** 1 The Higgs Centre for Theoretical Physics, University of Edinburgh, JCMB, KB, Mayfield Rd, Edinburgh, EH9 3JZ Scotland; 2 Rudolf Peierls Centre for Theoretical Physics, University of Oxford, 1 Keble Road, Oxford, OX1 3NP UK; 3 Theory Division, CERN, Geneva, Switzerland; 4 Dipartimento di Fisica, Università di Milano and INFN, Sezione di Milano, Via Veloria 16, 20133 Milan, Italy; 5 Dipartimento di Fisica, Università di Torino and INFN, Sezione di Torino, Via P. Giuria 1, 10125 Turin, Italy

## Abstract

We present an unbiased determination of the charm content of the proton, in which the charm parton distribution function (PDF) is parametrized on the same footing as the light quarks and the gluon in a global PDF analysis. This determination relies on the NLO calculation of deep-inelastic structure functions in the FONLL scheme, generalized to account for massive charm-initiated contributions. When the EMC charm structure function dataset is included, it is well described by the fit, and PDF uncertainties in the fitted charm PDF are significantly reduced. We then find that the fitted charm PDF vanishes within uncertainties at a scale $Q\sim 1.6$ GeV for all $x\lesssim 0.1$, independent of the value of $m_c$ used in the coefficient functions. We also find some evidence that the charm PDF at large $x\gtrsim 0.1$ and low scales does not vanish, but rather has an “intrinsic” component, very weakly scale dependent and almost independent of the value of $m_c$, carrying less than $1\%$ of the total momentum of the proton. The uncertainties in all other PDFs are only slightly increased by the inclusion of fitted charm, while the dependence of these PDFs on $m_c$ is reduced. The increased stability with respect to $m_c$ persists at high scales and is the main implication of our results for LHC phenomenology. Our results show that if the EMC data are correct, then the usual approach in which charm is perturbatively generated leads to biased results for the charm PDF, though at small *x* this bias could be reabsorbed if the uncertainty due to the charm mass and missing higher orders were included. We show that LHC data for processes, such as high $p_T$ and large rapidity charm pair production and $Z+c$ production, have the potential to confirm or disprove the implications of the EMC data.

## Introduction

Current general-purpose global PDF sets [1–7] assume that the charm PDF is perturbatively generated through pair production from gluons and light quarks. This assumption could be a limitation, and possibly a source of bias, for at least three different reasons. First, the charm PDF might have a non-vanishing “intrinsic” component of non-perturbative origin, such that it does not vanish at any scale within the perturbative region (see [8] for a recent review). Second, even if the charm PDF is purely perturbative in origin and thus vanishes below the physical threshold for its production, it is unclear what the value of this physical threshold is, as it is related to the charm pole mass, which in itself is not known very precisely. Finally, even if charm is entirely perturbative, and we knew accurately its production threshold, in practice massive charm production cross sections are only known at low perturbative order (at most NLO) and it is unclear whether this leads to sufficiently accurate predictions.

All these difficulties are solved if the charm quark PDF is parametrized and determined along with light quark and gluon PDFs. Whether or not the PDF vanishes, and, if it does, at which scale, will then be answered by the fit. From this point of view, the distinction between the perturbatively generated component, and a possible intrinsic component (claimed to be power suppressed [8, 9] before mixing with other PDFs upon perturbative evolution) becomes irrelevant. This is quite advantageous because the ensuing PDF set automatically incorporates in the standard PDF uncertainty the theoretical uncertainty related to the size of the perturbative charm component due to uncertainty in the value of the charm mass. Also, the possible intrinsic component, though concentrated at large *x* at a suitably chosen starting scale, will affect non-trivially PDFs at lower *x* at higher scale due to mixing through perturbative evolution.

The aim of this paper is to perform a first determination of the charm PDF of the proton in which no assumption is made about its origin and shape, and charm is treated on the same footing as the other fitted PDFs. This will be done using the NNPDF methodology: we will present a variant of the NNPDF3.0 [1] PDF determination, in which the charm PDF is parametrized in the same way as the light quark and gluon PDFs, i.e. with an independent neural network with 37 free parameters. In the present analysis, we will assume the charm and anticharm PDFs to be equal, since there is currently not data which can constrain their difference.

The possibility of introducing a non-perturbative “intrinsic” charm PDF has been discussed several times in the past; see e.g. Refs. [10–15]. In all of these earlier studies, only charm PDFs with a restrictive parametrization based on model assumptions are considered. Moreover, in the CT family of PDF determinations [11, 13, 15], intrinsic charm is introduced as a non-vanishing boundary condition to PDF evolution, but the massive corrections to the charm-initiated contributions [16, 17] are not included. While this would be consistent if all charm were generated perturbatively, as in the standard FONLL [18, 19] or S-ACOT [20] schemes, when there is a non-perturbative charm PDF it is justified only if this non-perturbative component is uniformly power-suppressed (of order $\Lambda ^2/m_c^2$, as in Ref. [21]) over the full range of *x*.

Here, however, as explained above, we wish to be able to parametrize the charm PDF at any scale, without committing ourselves to any specific hypothesis on its shape, and without having to separate the perturbative and non-perturbative components. A formalism which includes the mass corrections [16, 17] by extending the FONLL [18] GM-VFN scheme for deep-inelastic scattering of Ref. [19] was implemented at NLO [22] and consistently worked out to all orders in [23]. It is this implementation that will be used in this paper.

In the present PDF fit we use essentially the same data as in the NNPDF3.0 PDF determination, including as before the HERA charm production cross-section combination [24], but extended to also include the EMC charm structure function data of Ref. [25], which is the only existing measurement of the charm structure function at large *x*. We also replace all the HERA inclusive structure function data with the final combined dataset [5].

The outline of the paper is the following. First, in Sect. 2 we present the settings of the analysis: the dataset we use, the NLO implementation of the theory of Refs. [22, 23] for the inclusion of a fitted charm PDF, and the fit settings which have been used in the PDF fits.

In Sect. 3 we present the fit results: we compare PDF determinations with and without fitted charm; we discuss the stability of our results with respect to variations of the charm mass; and we discuss the features of our best-fit charm PDF, specifically in terms of the momentum fraction carried by charm, and in comparison to existing models. In Sect. 4 we discuss the implications of our results for LHC phenomenology, both for processes which are particularly sensitive to the charm PDF and thus might be used for its determination (such as $Z+c$ and charm pair production), and for LHC standard candles (such as *W*, *Z* and Higgs production). Finally, in Sect. 5 we discuss the delivery of our results and outline future developments.

## Settings

The PDF determination presented in this paper, which we will denote by NNPDF3IC, is based on settings which are similar to those used for the latest NNPDF3.0 global analysis [1], but with a number of differences, mostly related to the inclusion of a fitted charm PDF. These involve the experimental data, the theory calculations, and the fit settings, which we now discuss in turn.

### Experimental data

The dataset used in the present analysis is the same as used for NNPDF3.0, with two differences. The first has to do with HERA data: for NNPDF3.0, the combined inclusive HERA-I data [26] were used along with the separate HERA-II datasets from the H1 and ZEUS Collaborations [27–30]. Meanwhile, the final HERA legacy combination [5] data have become available. These have been used here. It has been shown [31] that, while the impact of the HERA-II data on top of the HERA-I combined data is moderate but not-negligible, the impact of the global legacy combination in comparison to HERA-I and separate HERA-II measurements is extremely small. Nevertheless, this replacement is performed for general consistency. Similar conclusions on the impact of these data have been reached by the MMHT group [32].

The second difference is that we will also include EMC charm structure function data [25]. Since the EMC Collaboration presented this measurement in the early 1980s, some studies [10, 12] have suggested that these data might provide direct evidence for non-perturbative charm in the proton [8, 33]. On the other hand, some previous PDF fits with intrinsic charm have not been able to provide a satisfactory description of this dataset [14]. Since it is known that the EMC measurements were affected by some systematic uncertainties which were only identified after the experiment was completed, we will perform fits both with and without it. We will also perform fits where the EMC charm data have been rescaled to match the current value of the branching ratio of charm quarks into muons.

Summarizing, the dataset that we will use is the following: fixed-target neutral-current deep-inelastic scattering (DIS) data from NMC [34, 35], BCDMS [36, 37], SLAC [38] and EMC [25]; the legacy HERA combinations for inclusive [5] and charm [24] reduced cross sections; charged-current structure functions from CHORUS inclusive neutrino DIS [39] and from NuTeV dimuon production data [40, 41]; fixed-target E605 [42] and E866 [43–45] Drell–Yan production data; Tevatron collider data including the CDF [46] and D0 [47] *Z* rapidity distributions and the CDF [48] one-jet inclusive cross sections; LHC collider data including ATLAS [49–51], CMS [52–55] and LHCb [56, 57] vector boson production, ATLAS [58, 59] and CMS [60] jets, and finally, total cross-section measurements for top quark pair production data from ATLAS and CMS at 7 and 8 TeV [61–66]. Data with $Q<3.5$ GeV and $W^2<12.5$ GeV$^2$ are excluded from the fit.

A final change in comparison to Ref. [1] is that we now impose additional cuts on the Drell–Yan fixed-target cross-section data:

1 $$\begin{aligned} \tau \le 0.08 , \quad |y|/y_\mathrm{max} \le 0.663 , \end{aligned}$$

where $\tau =M^2/s$ and $y_\mathrm{max}=- (1/2) \log \tau$, and *y* is the rapidity and *M* the invariant mass of the dilepton pair. These cuts are meant to ensure that an unresummed perturbative fixed-order description is adequate; the choice of values is motivated by studies performed in Ref. [67] in relation to the determination of PDFs with threshold resummation, which turns out to have a rather larger impact on Drell–Yan production than on deep-inelastic scattering. These cuts reduce by about a factor 2 the number of fixed-target Drell–Yan data points included here in comparison to Ref. [1], and they improve the agreement between theory and data.

### Theory

In the presence of fitted charm, the original FONLL expressions for deep-inelastic structure functions of Ref. [19] need to be modified to account for the new massive charm-initiated contributions [22, 23]. Also, while in previous NNPDF determinations pole quark masses only have been used, here we will consider both pole and $\overline{\mathrm{MS}}$ heavy quark masses. These new features have been implemented along with a major update in the codes used to provide the theory calculations. Indeed, in all previous NNPDF determinations, PDF evolution and the computation of deep-inelastic structure functions were performed by means of the Mellin-space FKgenerator NNPDF internal code [68, 69]. Here (and henceforth) we will use the public *x*-space APFEL code [70] for the solution of evolution equations and the computation of DIS structure functions. For hadronic observables, PDF evolution kernels are pre-convoluted with APPLgrid [71] partonic cross sections using the APFELcomb interface [72].

**Fig. 1:**
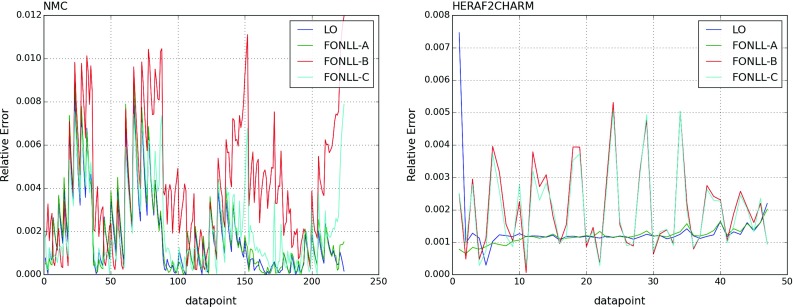
Representative benchmark comparisons between deep-inelastic structure functions computed with the FKgenerator and APFEL programs. We show the relative differences between the two codes for $\sigma _\mathrm{NC}^p(x,Q^2)$ at the NMC data points (*left*) and for $\sigma _\mathrm{c\bar{c}}(x,Q^2)$ for the HERA charm data points (*right*). In each case, we show results at LO (massless calculation) and for the FONLL-A, B and C general-mass schemes

The FKgenerator and APFEL codes have been extensively benchmarked. As an illustration, in Fig. 1 we show representative benchmark comparisons between deep-inelastic structure functions computed with the two codes. We plot the relative differences between the computation with either of these two codes of the inclusive neutral-current cross sections $\sigma _\mathrm{NC}(x,Q^2)$ at the NMC data points and for the charm production reduced cross sections $\sigma _\mathrm{c\bar{c}}(x,Q^2)$ for the HERA data points. In each case we compare results obtained at LO (massless calculation) and using the FONLL-A, B and C general-mass schemes. Similar agreement is found for all other DIS experiments included in NNPDF3.0.

The agreement is always better than 1%. Differences can be traced to the interpolation used by the FKgenerator, as demonstrated by the fact that they follow roughly the same pattern for all theoretical computations shown, with the largest differences observed for the NMC data, in the large *x*, low $Q^2$ region where the interpolation is most critical. Specifically, FKgenerator uses a fixed grid in *x* with 25 points logarithmically spaced in $[x=10^{-5},x=10^{-1}]$ and 25 points linearly spaced in $[x=10^{-1},x=1]$, while APFEL instead optimises the distribution of the *x*-grid points experiment by experiment. Hence we estimate that with the current APFEL implementation accuracy has significantly improved to better than 1%.

An advantage of using APFEL to compute DIS structure functions is that it allows for the use of either pole or $\overline{\mathrm{MS}}$ heavy quark masses [73, 74]. The implementation of running masses in the PDF evolution in APFEL has been benchmarked with the HOPPET program [75], finding better than 0.1% agreement. In addition, the APFEL calculation of structure functions with running heavy quark masses in the fixed three-flavour number scheme has been compared with the OpenQCDrad code [4], with which it has been found to agree at the 1% level.

**Fig. 2:**
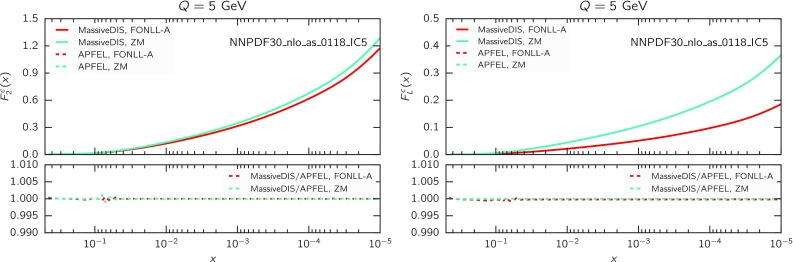
Benchmarking of the implementation in the APFEL and MassiveDISsFunction codes of deep-inelastic structure functions in the FONLL-A scheme with intrinsic charm of Refs. [22, 23]. The charm structure functions $F_2^c(x,Q^2)$ (*left*) and $F_L^c(x,Q^2)$ (*right*) are shown as a function of *x* for $Q=5$ GeV; the relative difference between the two codes is shown in the *lower panel*. In each case we show full matched FONLL-A results as well as the purely massless calculation

Massive charm-initiated terms for both neutral and charged-current processes have been implemented in APFEL up to $\mathcal {O}\left( \alpha _s\right)$. Target mass corrections are included throughout. The implementation has been validated through benchmarking against the public stand-alone MassiveDISsFunction code,[76] which also implements the theory calculations of Refs. [22, 23]. Some illustrative comparisons between the charm structure functions $F_2^c(x,Q^2)$ and $F_L^c(x,Q^2)$, computed using APFEL and MassiveDISsFunction, are shown in Fig. 2. The various inputs to the FONLL-A scheme computation, namely the three- and four-flavour scheme results are shown, along with the full matched result, as a function of *x* at the scale $Q=5$ GeV, computed using an input toy intrinsic charm PDF, corresponding to the NNPDF30_nlo_as_0118_IC5 set of Ref. [22]. The two codes turn out to agree at the 0.1% level or better, for all neutral-current and charged-current structure functions.

### Fit settings

We can now specify the theory settings used for the PDF fits presented in this paper. We will use NLO theory with $\alpha _s(M_Z)=0.118$, with a bottom mass of $m_b=4.18$ GeV. We will present fits with the $\overline{\mathrm{MS}}$ charm mass set equal to $m_c(m_c)=1.15,\> 1.275$ and 1.40 GeV, which corresponds to the PDG central value and upper and lower five-sigma variations [77]. We will also present fits with the charm pole mass $m_c^\mathrm{pole}=1.33$, 1.47 and 1.61 GeV, obtained from the corresponding $\overline{\mathrm{MS}}$ values using one-loop conversion. This conservative range of charm pole mass value allows us to account for the large uncertainties in the one-loop conversion factor. In addition, as a cross-check, we also perform a pole mass fit with $m_c^\mathrm{pole}=1.275$ GeV, which was the choice adopted in NNPDF3.0. When the charm PDF is generated perturbatively, the charm threshold is set to be the charm mass. The input parametrization scale is $Q_0=1.1$ GeV for the fits with perturbative charm and $Q_0=1.65$ GeV in the case of fitted charm, ensuring that the scale where PDFs are parametrized is always above (below) the charm threshold for the analysis with fitted (perturbative) charm in all the range of charm masses considered. In sum, we will consider seven charm mass values (four pole, and three $\overline{\mathrm{MS}}$), and for each of them we will present fits with perturbative charm or with fitted charm.

In the NNPDF3.0 analysis, seven independent PDF combinations were parametrized with artificial neural networks at the input evolution scale $Q_0$: the gluon, the total quark singlet $\Sigma$, the non-singlet quark triplet and octet $T_3$ and $T_{8}$ and the quark valence combinations *V*, $V_3$ and $V_8$. In this analysis, when we fit the charm PDF, we use the same PDF parametrization basis supplemented by the total charm PDF $c^+$, that is,

2 $$\begin{aligned} c^+(x,Q_0)\equiv c(x,Q_0)+\bar{c}(x,Q_0) = x^{a_{c^+}}(1-x)^{b_{c^+}}\mathrm{NN}_{c^+}(x) , \end{aligned}$$

with $\mathrm{NN}_{c^+}(x)$ a feed-forward neural network with the same architecture (2-5-3-1) and number of free parameters (37) as the other PDFs included in the fit, and $a_{c^+}$ and $b_{c^+}$ the corresponding preprocessing exponents, whose range is determined from an iterative procedure designed to ensure that the resulting PDFs are unbiased. In addition, we assume that the charm and anticharm PDFs are the same, $c^-(x,Q_0)\equiv c(x,Q_0)-\bar{c}(x,Q_0)=0$. Since at NLO this distribution evolves multiplicatively, it will then vanish at all values of $Q^2$. It might be interesting to relax this assumption once data able to constrain $c^-(x,Q_0)$ become available.

The fitting methodology used in the present fits is the same as in NNPDF3.0, with some minor improvements. First, we have enlarged the set of positivity constraints. In NNPDF3.0, positivity was imposed for the up, down and strange structure functions, $F_2^u$, $F_2^d$ and $F_2^s$; for the light component of the longitudinal structure function, $F_L^l$; and for Drell–Yan rapidity distributions with the flavour quantum numbers of $u\bar{u}$, $d\bar{d}$, and $s\bar{s}$; and for the rapidity distribution for Higgs production in gluon fusion (see Section 3.2.3 of Ref. [1] for a detailed discussion). This set of positivity observables has now been enlarged to also include flavour non-diagonal combinations: we now impose the positivity of the *ud*, $\bar{u}d$, $\bar{u}\bar{d}$ and $u\bar{d}$ Drell–Yan rapidity distributions. As in Ref. [1], positivity is imposed for all replicas at $Q^2_\mathrm{pos}=5$ GeV$^2$, which ensures positivity for all higher scales.

Also, we have modified the way asymptotic exponents used in the iterative determination of the preprocessing range are computed. Specifically, we now use the definition

3 $$\begin{aligned} \alpha _{f_i}(x,Q^2) \equiv \frac{\partial \ln [xf_i(x,Q^2)]}{\partial \ln x} \ \ \ \ \ \ \ \, \beta _{f_i}(x,Q^2) \equiv \frac{\partial \ln [xf_i(x,Q^2)]}{\partial \ln (1-x)} \,\text{, } \end{aligned}$$

suggested in Ref. [78, 79], which is less affected by sub-asymptotic terms at small and large-*x* than the definition used in the NNPDF3.0 analysis [1]. This allows a more robust determination of the ranges in which the PDF preprocessing exponents should be varied, following the iterative procedure discussed in [1]. This modification affects only the PDFs in the extrapolation regions where there are little or none experimental data constraints available. The implications of these modifications in the global analysis will be more extensively discussed in a forthcoming publication.

## Results

In this section we discuss the main results of this paper, namely the NNPDF3 PDF sets with fitted charm. After presenting and discussing the statistical indicators of the fit quality, we discuss the most significant effects of fitted charm, namely, its impact on the dependence of PDFs on the charm mass, and its effect on PDF uncertainties. We then discuss the extent to which our results are affected by the inclusion of EMC data on the charm structure function. Having established the robustness of our results, we turn to a study of the properties of the fitted charm PDF: whether or not it has an intrinsic component, the size of the momentum fraction carried by it, and how it compares to some of the models for intrinsic charm constructed in the past.

Here and henceforth we will refer to a fit using the FONLL-B scheme of Ref. [19], in which all charm is generated perturbatively, both at fixed order and by PDF evolution, as “perturbative charm”, while “fitted charm” refers to fit obtained using the theory reviewed in Sect. 2.2. Note that fitted charm includes a perturbative component, which grows above threshold until it eventually dominates: at high enough scales most charm is inevitably perturbative. However, close to threshold the non-perturbative input might still be important: in particular below threshold the perturbative charm vanishes by construction, whereas the fitted charm can still be non-zero (so-called “intrinsic” charm).

**Table 1:** Statistical estimators of the fitted and perturbative charm PDFs for the central value of the charm pole mass, for both fitted charm and perturbative charm. For $\chi ^2$ and $\left\langle \chi ^2\right\rangle$ we provide the results using both the $t_0$ and the “experimental” definition of the $\chi ^2$ (see text). $\left\langle E_\mathrm{tr}\right\rangle$ and $\left\langle E_\mathrm{val}\right\rangle$ are computed during the fit using the $t_0$ definition.

NNPDF3 NLO $m_c=1.47$ GeV (pole mass)		
	Fitted charm	Perturbative charm
$\chi ^2/N_\mathrm{dat}$ (exp)	1.159	1.176
$\left\langle \chi ^2\right\rangle _\mathrm{rep}/N_\mathrm{dat}$ (exp)	$1.40\pm 0.24$	$1.33\pm 0.12$
$\chi ^2/N_\mathrm{dat}$ ($t_0$)	1.220	1.227
$\left\langle \chi ^2\right\rangle _\mathrm{rep}/N_\mathrm{dat}$ ($t_0$)	$1.47 \pm 0.26$	$1.38 \pm 12$
$\left\langle E_\mathrm{tr} \right\rangle /N_\mathrm{dat}$	$2.38 \pm 0.29$	$2.32 \pm 0.16$
$\left\langle E_\mathrm{val} \right\rangle /N_\mathrm{dat}$	$2.60 \pm 0.37$	$2.48 \pm 0.16$
$\left\langle \mathrm{TL} \right\rangle$	$\left( 3.5 \pm 0.8\right) \times 10^3$	$\left( 2.2 \pm 0.8\right) \times 10^3$
$\varphi _{\chi ^2}$	$0.49 \pm 0.02$	$0.40\pm 0.01$
$\left\langle \sigma ^\mathrm{(exp)}\right\rangle _\mathrm{dat}$	13.1%	12.2%
$\left\langle \sigma ^\mathrm{(fit)}\right\rangle _\mathrm{dat}$	7.4%	4.4%

**Table 2:** The $\chi ^2$ per data point for the experiments included in the present analysis, computed using the experimental covariance matrix, comparing the results obtained with fitted charm with those of perturbative charm. We also provide the total $\chi ^2/N_\mathrm{dat}$ of the fit, as well as the number of data points per experiment. In the case of perturbative charm, we indicate the values of the fit without the EMC data, and show in brackets the $\chi ^2$ of this experiment when included in the fit.

NNPDF3 NLO $m_c=1.47$ GeV (pole mass)			
Experiment	$N_\mathrm{dat}$	$\chi ^2/{N_\mathrm{dat}}$	$\chi ^2/{N_\mathrm{dat}}$
		Fitted charm	Perturbative charm
NMC	325	1.36	1.34
SLAC	67	1.21	1.32
BCDMS	581	1.28	1.29
CHORUS	832	1.07	1.11
NuTeV	76	0.62	0.62
EMC	16	1.09	[7.3]
HERA inclusive	1145	1.17	1.19
HERA charm	47	1.14	1.09
DY E605	104	0.82	0.84
DY E866	85	1.04	1.13
CDF	105	1.07	1.07
D0	28	0.64	0.61
ATLAS	193	1.44	1.41
CMS	253	1.10	1.08
LHCb	19	0.87	0.83
$\sigma (t\bar{t})$	6	0.96	0.99
Total	3866	1.159	1.176

### Fit results

In Tables 1 and 2 we collect the statistical estimators for our best fit with central value of the charm pole mass, namely $m_c^\mathrm{pole}=1.47$ GeV, both with fitted and perturbative charm. A detailed discussion of statistical indicators and their meaning can be found in Refs. [1, 69, 80, 81]. Here we merely recall that $\chi ^{2}$ is computed by comparing the central (average) fit to the original experimental data; $\left\langle \chi ^{2} \right\rangle _\mathrm{rep}$ is computed by comparing each PDF replica to the data and averaging over replicas, while $\left\langle E \right\rangle$ is the quantity that is actually minimized, i.e. it coincides with the $\chi ^{2}$ computed by comparing each replica to the data replica it is fitted to, with the two values given corresponding to the training and validation datasets, respectively. The values of $\left\langle E \right\rangle$ are computed using the so-called $t_0$ definition of the $\chi ^2$, while for $\chi ^2$ and $\left\langle \chi ^{2} \right\rangle _\mathrm{rep}$ we show in the table values computed using both the $t_0$ and the “experimental” definition (see Refs. [82, 83] for a discussion of different $\chi ^2$ definitions); they are seen to be quite close anyway.

Moreover, $\langle \mathrm{TL}\rangle$ is the training length, expressed in number of cycles (generations) of the genetic algorithm used for minimization. $\varphi _{\chi ^2}$ [1] is the average over all data of uncertainties and correlations normalized to the corresponding experimental quantities (i.e., roughly speaking, $\varphi _{\chi ^2}=0.5$ means that the PDF uncertainty is half the uncertainty in the original data), while $\left\langle \sigma ^\mathrm{(exp)}\right\rangle _\mathrm{dat}$ is the average percentage experimental uncertainty, and $\left\langle \sigma ^\mathrm{(fit)}\right\rangle _\mathrm{dat}$ is the average percentage PDF uncertainty at data points.

In Table 2 we provide a breakdown of the $\chi ^2$ per data point for all experiments (the value computed with the “experimental” definition only). In the case of perturbative charm, the $\chi ^2$ values listed correspond to a fit without EMC data, with the $\chi ^2$ for this experiment if it were included in the fit given in square parentheses. Note that the total $\chi ^2$ values in this table are significantly lower than those reported in our previous global fit NNPDF3.0 [1]: this is mainly due the much lower $\chi ^2$ value for HERA data, which in turn results from using the full combined HERA dataset instead of separate HERA-II H1 and ZEUS data.

**Fig. 3:**
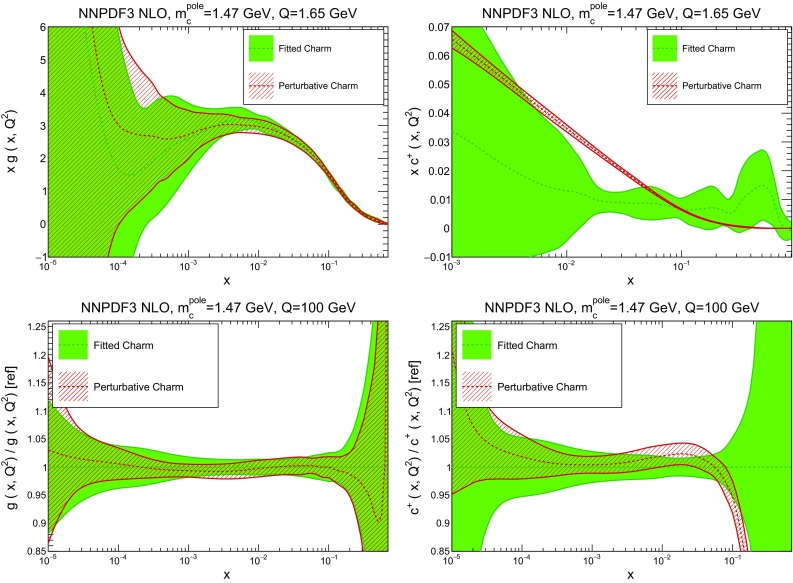
Comparison of the NNPDF3 NLO PDFs with fitted and perturbative charm, for a charm pole mass of $m^\mathrm{pole}_c=1.47$ GeV. We show the gluon (*left plots*) and the charm quark (*right plot*), at a low scale $Q=1.65$ GeV (*upper plots*) and at a high scale, $Q=100$ GeV (*lower plots*). In the latter case, results are shown normalized to the central value of the fitted charm PDFs

**Fig. 4:**
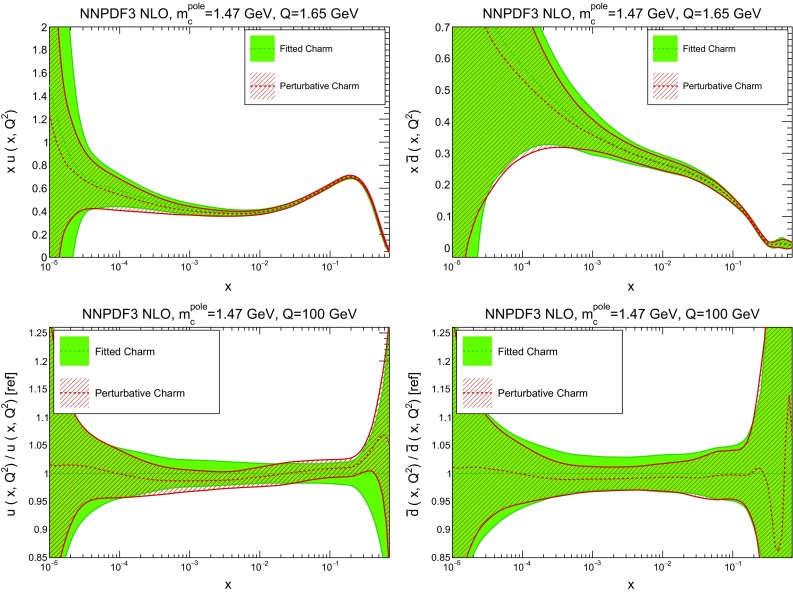
Same as Fig. 3, but now showing the up (*left*) and antidown (*right*) PDFs

It is clear from these comparisons that fitting charm has a moderate impact on the global fit: the fit is somewhat longer (by less than two sigma), and uncertainties on predictions are a little larger. However, the overall quality of the global fit is somewhat improved: at the level of individual experiments, in most cases the fit quality is similar, with the improvements in the case of fitted charm more marked for the HERA inclusive, SLAC, CHORUS and E866 data. The $\chi ^2/N_\mathrm{dat}$ of the HERA charm combination is essentially the same in the fitted and perturbative charm cases, and the fit quality to the LHC experiments is mostly unaffected, as expected since the measurements included have very limited direct sensitivity to the charm PDF.

On the other hand, the EMC charm structure function data cannot be fitted in a satisfactory way with perturbative charm: the best we can do without fitted charm is $\chi ^2/N_\mathrm{dat}=7.3$, corresponding to an increase in the total $\chi ^2$ of over 100 units. However, the $\chi ^2$ to these data improves dramatically when charm is fitted, and an excellent description with $\chi ^2/N_\mathrm{dat}=1.09$ is achieved. It is interesting to note that some previous PDF determinations with intrinsic charm had difficulties in providing a satisfactory description of the EMC charm structure function data (see e.g. Ref. [14]). In the following, the EMC charm data will be excluded from the default fits with perturbative charm, though we will come back to the issue of including these data when charm is purely perturbative when discussing charm mass dependence in Sect. 3.2, and when specifically analyzing the impact of these data in Sect. 3.3.

In Figs. 3 and 4 we compare several PDFs with fitted and perturbative charm, both at a low scale, $Q=1.65$ GeV (just above the scale at which charm is generated in the purely perturbative fit), and at a high scale, $Q=100$ GeV. It is clear that light quarks and especially the gluon are moderately affected by the inclusion of fitted charm, with a barely visible increase in the PDF uncertainty. The charm PDF and especially its uncertainty are affected more substantially: we will discuss this in detail in Sect. 3.4.

### Dependence on the charm quark mass and fit stability

As discussed in the introduction, one of the motivations for introducing a fitted charm PDF is to separate the role of the charm mass as a physical parameter from its role in determining the boundary condition of the charm PDF. This dual role, played by the charm mass, can be disentangled by studying the dependence of the fit results (and in particular the charm PDF) on the value of the charm mass when charm is perturbative or fitted. To this purpose, we compare fit results obtained when the charm mass is varied between $m_c^\mathrm{pole}=1.33$ and 1.61 GeV about our central $m_c^\mathrm{pole}=1.47$, corresponding to a five-sigma variation in units of the PDG uncertainty on the $\overline{\mathrm{MS}}$ mass $m_c(m_c)$ using one-loop conversion to pole. After examining the stability of our results on the charm mass value, we discuss their stability with respect to different theoretical treatments. First, we show results for a fit with $m_c^\mathrm{pole}=1.275$ GeV, produced in order to compare with a fit with $\overline{\mathrm{MS}}$ masses with the same numerical value of $m_c$, and then we discuss how the fit results change if we switch from pole to $\overline{\mathrm{MS}}$ masses. Finally, we discuss how fit results would change if an S-ACOT-like treatment of the heavy quark was adopted, in which massive corrections to charm-initiated contributions are neglected.

**Fig. 5:**
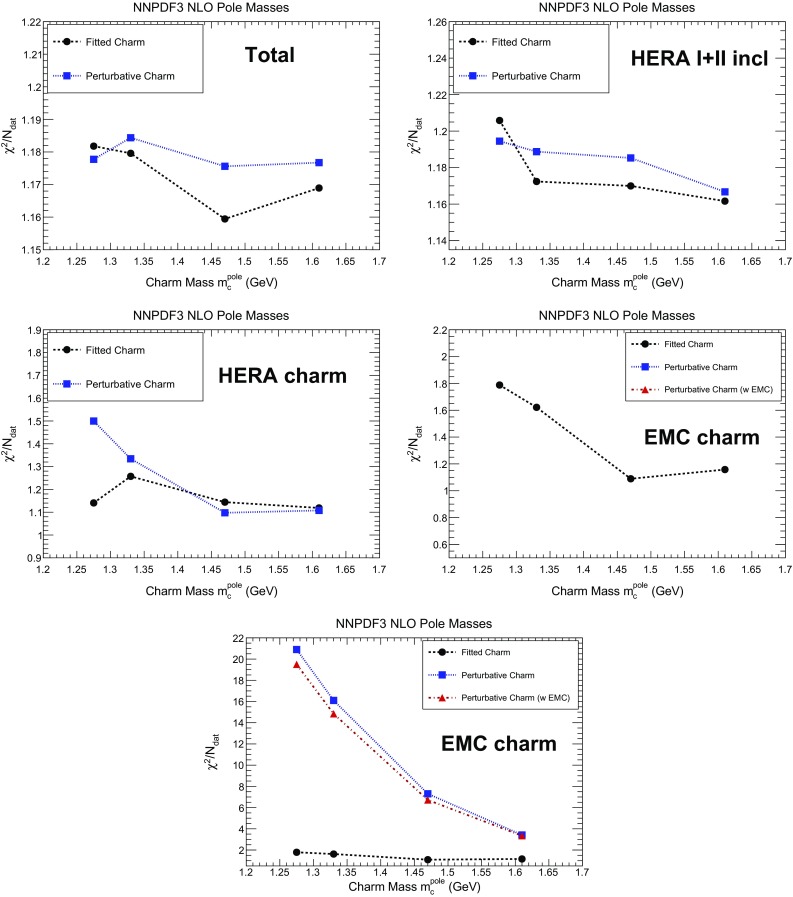
The $\chi ^2$ per data point for the total dataset (*top left*); for the HERA inclusive (*top right*) and charm structure function (*center left*) combined datasets and for the EMC charm data (*center right*), for fits with perturbative and fitted charm, as a function of the value of the charm pole mass $m_c^\mathrm{pole}$. In the *bottom row* the $\chi ^2$ for the EMC charm data is shown again with an enlarged scale which enables the inclusion of the values for perturbative charm; in this plot only for fits with perturbative charm we show results both with and without the EMC data included in the fit. In all other plots, the perturbative charm results are for fits without EMC data. The fitted charm fits always include the EMC data

For a first assessment of the relative fit quality, in Fig. 5 we show $\chi ^2/N_\mathrm{dat}$ as a function of the pole charm mass value, in the fits both with perturbative and fitted charm. The plot has been produced using the experimental definition of the $\chi ^2$. The values shown here correspond to the full dataset, the inclusive and charm HERA structure function combined data, and EMC structure function data. In the case of perturbative charm, we generally show the results of a fit in which the EMC data are not included, except in the plot of the $\chi ^2$ to the EMC data themselves, where we show both fits with EMC data included and not included. It is seen that the EMC data cannot be fitted when charm is perturbative in the sense that their poor $\chi ^2$ does not significantly improve upon their inclusion in the fit. We will accordingly henceforth exclude the EMC data from all fits with perturbative charm, as their only possible effect would be to distort fit results without any significant effect on the fit quality.

It is interesting to observe that, while with fitted charm the EMC data seem to favour a value of the charm mass around 1.5 GeV, close to the current PDG average, with perturbative charm they would favour an unphysically large value. These results also suggest that a determination of the charm mass from a global fit with fitted charm might in principle be possible, but that this requires high statistics and precision analysis techniques, such as those used in Refs. [84, 85] for the determination of the strong coupling $\alpha _s$.

**Fig. 6:**
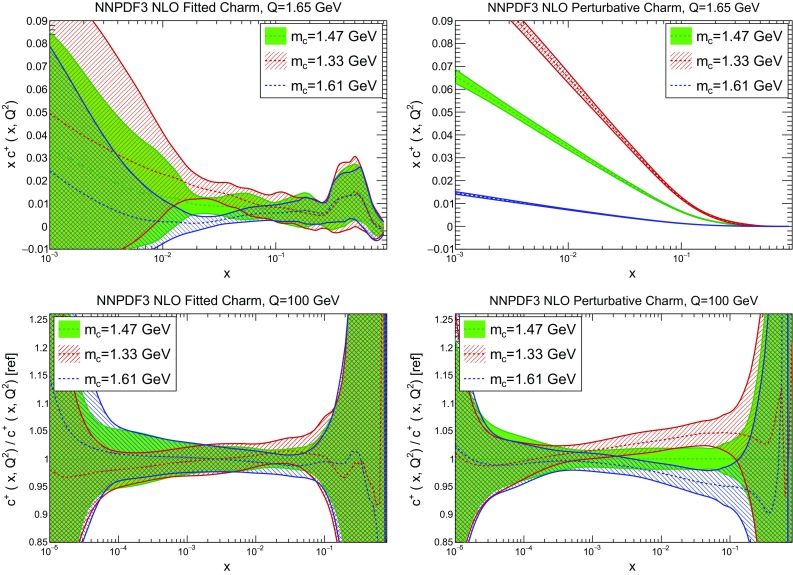
Dependence of the charm PDF on the value of the pole charm mass $m_c^\mathrm{pole}$: the charm PDF obtained with fitted charm (*left*) and perturbative charm (*right*) are compared for $m_c^\mathrm{pole}=1.33,~1.47$ and 1.61 GeV, at a low scale $Q=1.65$ GeV (*top*) and at a high scale $Q=100$ GeV (*bottom*). At high scale, PDFs are shown as a ratio to the fit with central $m_c^\mathrm{pole}=1.47$ GeV

**Fig. 7:**
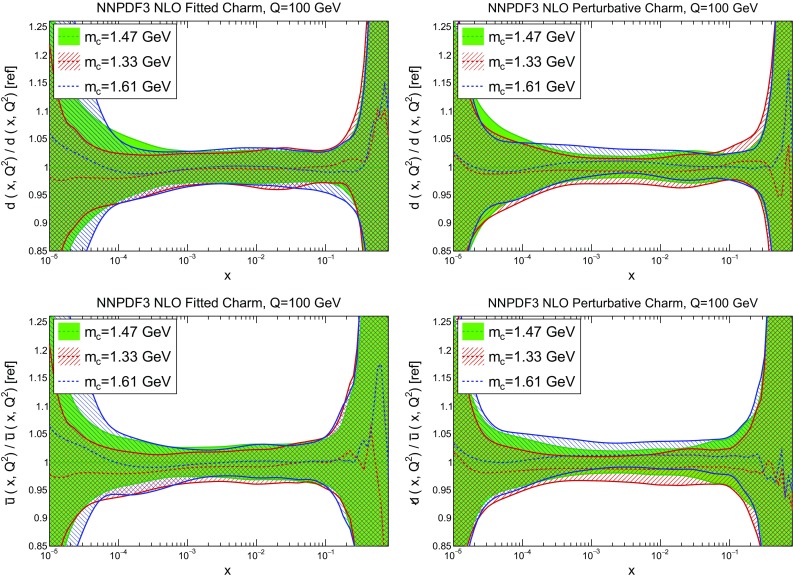
Same as the bottom row of Fig. 6, but now for the down (*top*) and antiup (*bottom*) PDFs

We now compare the PDFs obtained with different values of the charm mass both with perturbative and fitted charm: in Fig. 6 we show gluon and charm, and in Fig. 7 up and antidown quarks. Results are shown at low and high scale (respectively, $Q=1.65$ GeV and $Q=100$ GeV) for charm, and at a high scale only for the light quarks. Of course, with perturbative charm the size of the charm PDF at any given scale depends significantly on the value of the charm mass that sets the evolution length: the lower the mass, the lower the starting scale, and the larger the charm PDF at any higher scale. The percentage shift of the PDF as the mass is varied is of course very large close to threshold, but it persists as a sizeable effect even at high scale. Remarkably, this dependence all but disappears when charm is fitted: both at low and high scale the fitted charm PDF is extremely stable as the charm mass is varied. This means that indeed once charm is fitted, its size is mostly determined by the data, rather than by the (possibly inaccurate) value at which we set the threshold for its production. Interestingly, the other PDFs, and specifically the light quark PDFs, also become generally less dependent on the value of the heavy quark masses, even at high scale, thereby making LHC phenomenology somewhat more reliable.

**Fig. 8:**
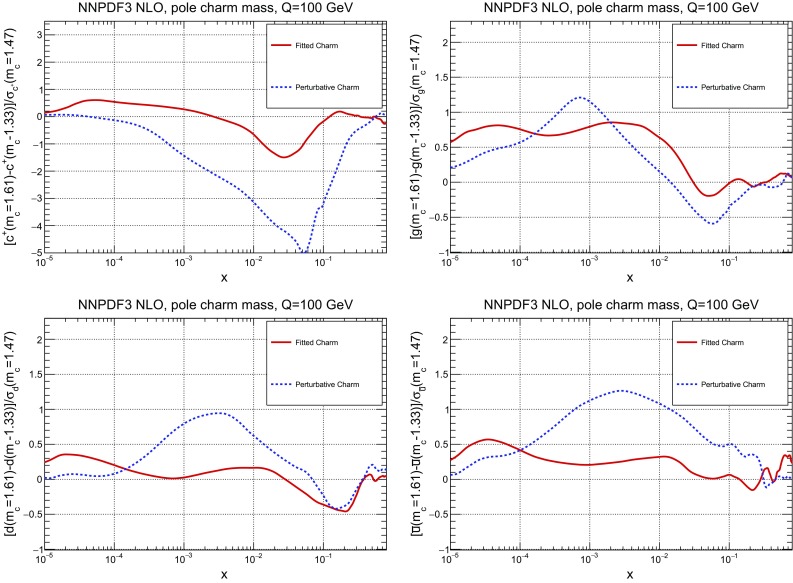
The pull Eq. (4) between PDFs determined with the two outer values of the quark mass ($m_c=1.61;\>1.33$ GeV), in units of the PDF uncertainty plotted as a function of *x* at $Q=100$ GeV. Results are shown for charm (*top left*), gluon (*top right*), down (*bottom left*) and antiup (*bottom right*). Note the different scale on the *y* axis in the different plots

This improved stability upon heavy quark mass variation can be seen in a more quantitative way by computing the pulls between the PDFs obtained using the two outer values of the charm mass, defined as

4 $$\begin{aligned} P_q(x,Q^2) \equiv \frac{q(x,Q^2)\vert _{m_{c}=1.61~\mathrm{GeV}} - q(x,Q^2)\vert _{m_{c}=1.33~\mathrm{GeV}}}{ \sigma _q(x,Q^2)\vert _{m_c=1.47~\mathrm{GeV}}} , \end{aligned}$$

where *q* stands for a generic PDF flavour, and $\sigma _q$ is the PDF uncertainty on the fit with the central $m_c$ value. The pull Eq. (4) evaluated at $Q=100$ GeV is plotted in Fig. 8 as a function of *x* for the charm, gluon, down and antiup PDFs. It is clear that once charm is fitted the pull is essentially always less than one (that is, the PDF central value varies by less than one sigma when the mass is varied in the given range), while it is somewhat larger for light quarks and gluon, and much larger (up to five sigma) for the charm PDF if charm is purely perturbative. The smallest difference is seen for the gluon, for which the pull is less than one in both cases, and in fact slightly larger for fitted charm when $x\sim 10^{-2}$.

**Fig. 9:**
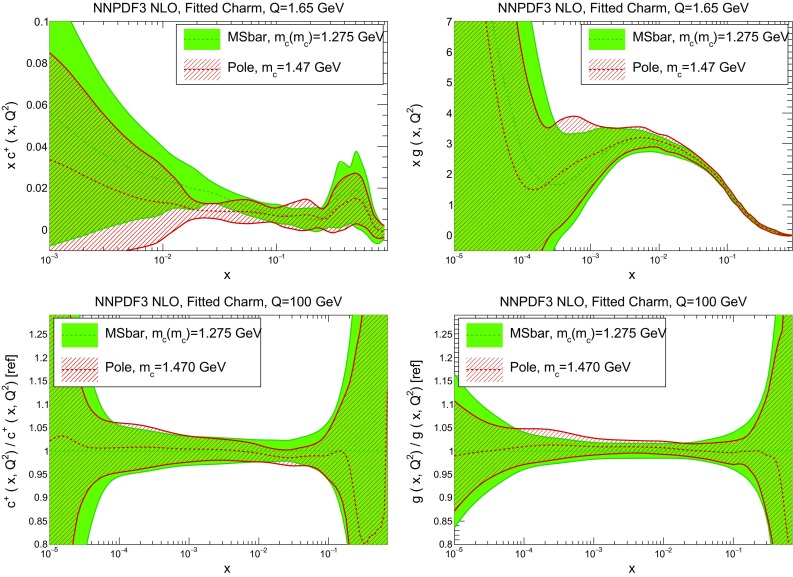
Comparison of PDFs determined with ${\overline{\mathrm {MS}}}$ vs. pole mass, for corresponding values of the mass obtained by one-loop conversion: $m_c^\mathrm{pole}=1.47$ GeV and $m_c(m_c)=1.275$ GeV. The charm (*left*) and gluon (*right*) PDFs are shown, at low scale $Q=1.65$ GeV (*top*) and high scale $Q=100$ GeV (*bottom*). In the high-scale plots, results are shown as a ratio to the ${\overline{\mathrm {MS}}}$ mass result

We next check the impact of switching from pole to $\overline{\mathrm {MS}}$ masses. In Fig. 9 we compare PDFs obtained using pole mass $m_c^\mathrm{pole}=1.47$ GeV, or $\overline{\mathrm {MS}}$ mass $m_c(m_c)=1.275$ GeV, the two values being related by one-loop perturbative conversion. The charm and gluon PDFs are shown, at low and high scale. It is clear that the change in results is compatible with a statistical fluctuation. Similar results hold for other PDFs.

**Fig. 10:**
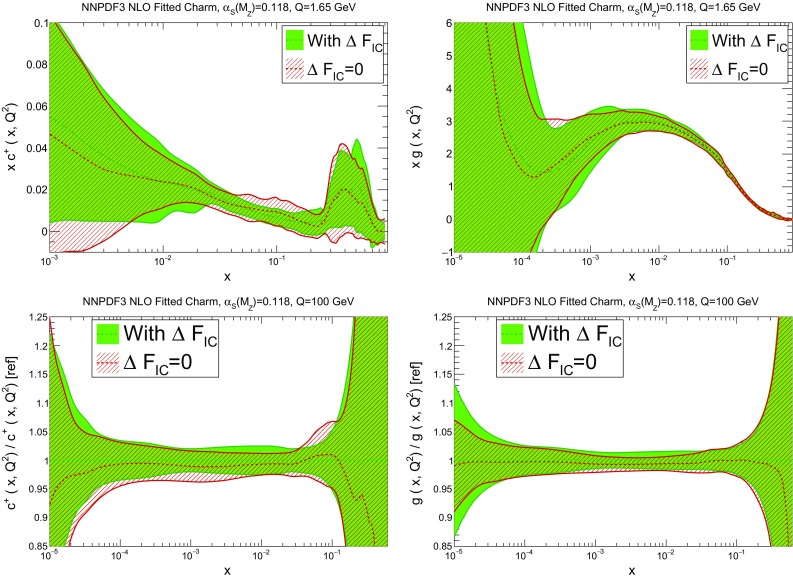
Same as Fig. 9, but now comparing our default results to the case in which massive charm-initiated contributions are neglected (original FONLL-B of Ref. [19] or S-ACOT; see *text*)

Finally, we study how our results would change if massive charm-initiated contributions are neglected, i.e., if the original FONLL-B scheme of Ref. [19] is used. This corresponds to setting to zero the correction term $\Delta F_h$ (Eq. (11) of Ref. [22]), it is [22, 23] completely equivalent to the S-ACOT scheme used in intrinsic charm studies by the CT Collaboration [11, 13, 15], and, as mentioned in the introduction, it might be justified if the intrinsic charm contribution is power-suppressed. Results are shown in Fig. 10: again, the change in results is compatible with a statistical fluctuation. This fact has some interesting implications. First, it shows that the size our best-fit charm is moderate, and compatible with a power-suppressed intrinsic charm. Also, it suggests that the approximate NNLO treatment of fitted charm proposed in Ref. [22], in which these terms are actually only included up to NLO (given that the massive charm-initiated coefficient functions are only known to this order [16, 17]), should actually be quite reliable. Finally, it should be noted that for the charm-initiated contribution the charm production threshold is set by $m_c$, but for the overall process, including the proton remnant, the threshold is set by $2m_c$, so there must be non-perturbative contributions which restore momentum conservation: these would appear as power-suppressed corrections which should be resummed to all orders when $W^2\sim m_c^2$. In our case $W^2\gg m_c^2$ for all *x*, and the charm-initiated contribution is seen to be sufficiently small, so that this issue should be of no concern.

### Impact of the EMC data

**Fig. 11:**
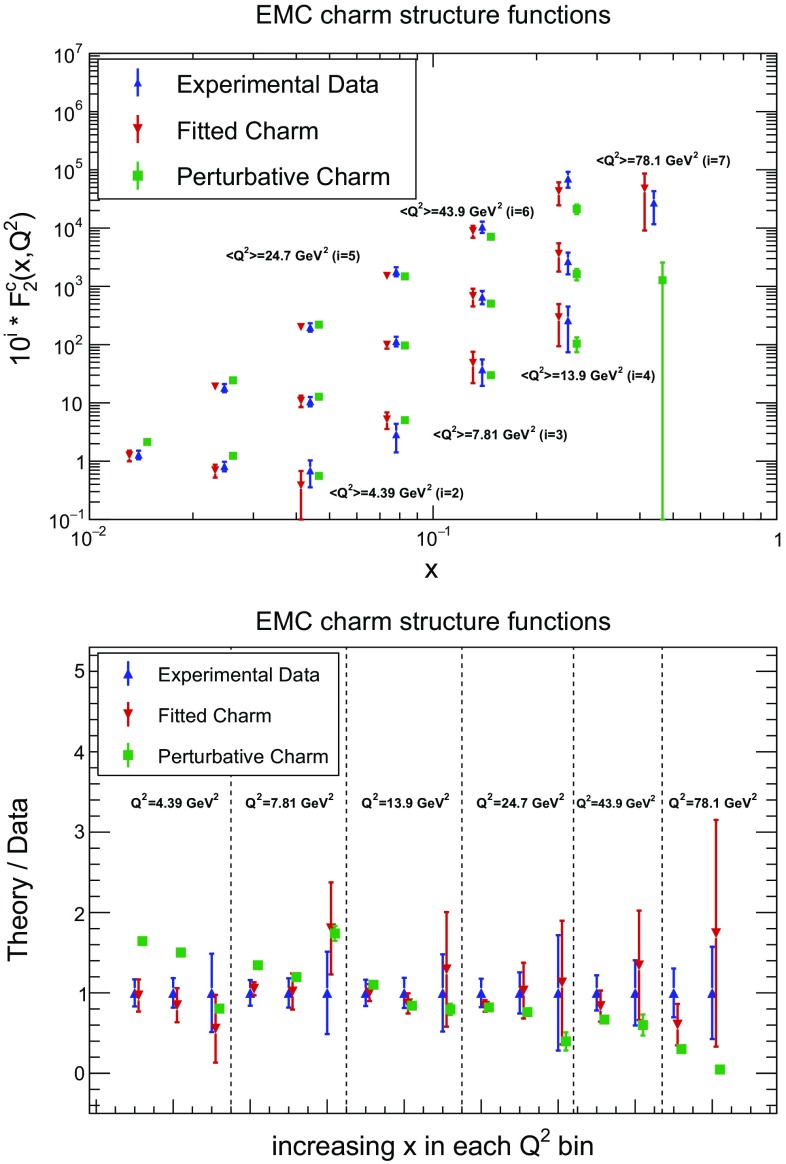
Comparison of the best-fit theoretical result to the experimental result for the EMC $F_2^c$ structure function data with fitted and with perturbative charm. The uncertainties shown are the total PDF uncertainty in each data bin for theory, and the total experimental uncertainty for the data. We show both the data vs. *x* in $Q^2$ bins, offset to improve readability (*top*), and the ratio of theory to data (*bottom*): here the order in each bin is from small to large values of *x*

As already noted, it is not possible to fit the EMC $F_2^c$ data of Ref. [25] with perturbative charm. It is then important to assess carefully the effect of these data when we fit charm. The purpose of this assessment is twofold. First, we have the phenomenological goal of assessing to which extent conclusions may be affected if the EMC data are entirely or in part unreliable, or perhaps have underestimated uncertainties. Second, perhaps more interestingly, we would like to understand whether, quite independently of the issue of their reliability, the EMC data might provide a realistic scenario in which not fitting charm would lead to biased fit results.

The agreement between data and theory when charm is fitted is illustrated in Fig. 11, where we compare the EMC charm structure function data with the structure function computed using the best-fit PDFs, with either fitted or perturbative charm. Both the absolute structure function (top) and the theory to data ratio (bottom) are shown. It is interesting to observe that the discrepancy between the data and the perturbative charm PDFs is large, and it is not confined in any specific region of *x* or $Q^2$, making an explanation of the discrepancy based on a single cause such as resummation or higher-order corrections rather unlikely. More specifically, it is clear that the data at large *x* in the highest $Q^2$ bins cannot be reproduced by perturbative charm, which gives a very small contribution in this region. Interestingly, in this region one has $Q^2\gtrsim 25$ GeV$^2$, so a possible higher-twist component that might imitate the charm contribution [86] would be quite suppressed. Likewise, in the small *x* region, $x\lesssim 0.1$, perturbative charm overshoots the data. Here again, higher twist is expected to be small since, although $Q^2$ is quite low, $W^2 \gtrsim 50$ GeV$^2$. The fitted charm PDF corrects both these discrepancies rather neatly, by increasing the charm content at large *x*, and reducing it at small *x*, to produce a perfectly satisfactory fit. This leads to the perhaps surprising conclusion that in order to fit the EMC data both a large-*x* positive bump (possibly of non-perturbative origin), and a small *x* undershoot (possibly mimicking missing higher-order corrections) are needed. Both the way the large *x* behaviour of our best-fit charm compares to existing models and its small *x* component compares to what we expect from missing higher orders will be discussed in Sect. 3.4 below.

**Fig. 12:**
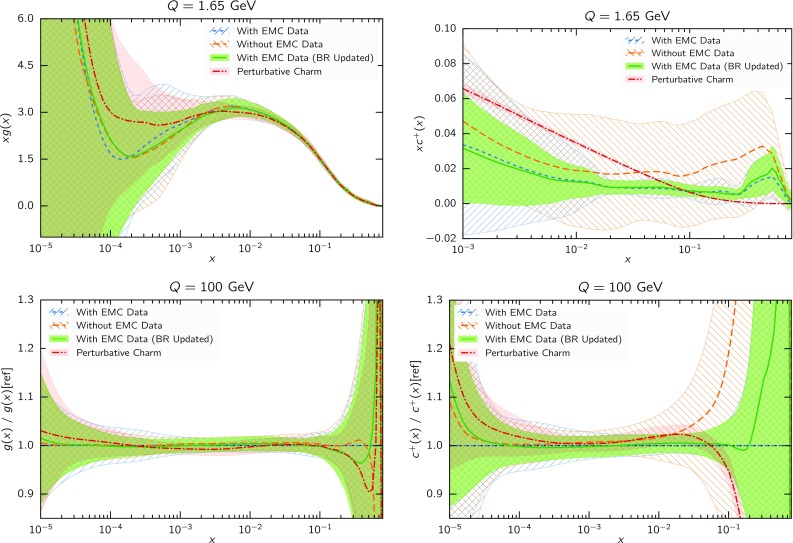
Same as Fig. 3, but now, when charm is fitted, also showing results obtained when EMC data are rescaled to match updated branching fraction of *D* mesons into muons (see *text*), or excluded altogether

The impact of the EMC data on the PDFs is illustrated in Fig. 12, where we compare the charm and gluon PDFs with and without the EMC data included in the fit, everything else being unchanged, with the perturbative charm fit also being shown for reference. It is clear that for all $x\gtrsim 10^{-2}$ the uncertainty on the fitted charm PDF is greatly increased in the absence of the EMC data. Reassuringly, the qualitative features of the central charm PDF (to be discussed more extensively in Sect. 3.4 below) do not change substantially: in particular it is still true that the central PDF at large *x* displays a bump, while at small *x* it lies below the perturbatively generated charm—though uncertainties are now so large that neither effect can be considered statistically significant. The other PDFs change very little.

We now specifically address the phenomenological issue of the reliability of the EMC data. First of all, it should be noticed that the published uncertainty in the EMC data is quite large to begin with: the average uncertainty is about 27%. This said, various issues have been raised concerning this dataset. Firstly, the inclusive EMC structure function data are known to be inconsistent with BCDMS data (see e.g. [87]), but this was due to underestimated backgrounds in drift chambers. Therefore, this problem is expected to be absent in the charm structure function data which were taken with a calorimetric target [88]. The correction is anyway never more than 20% [87], hence much smaller than the effect seen in Fig. 11. In Ref. [89] it was checked explicitly that if the inclusive EMC data are added to the fit they have essentially no impact.

**Fig. 13:**
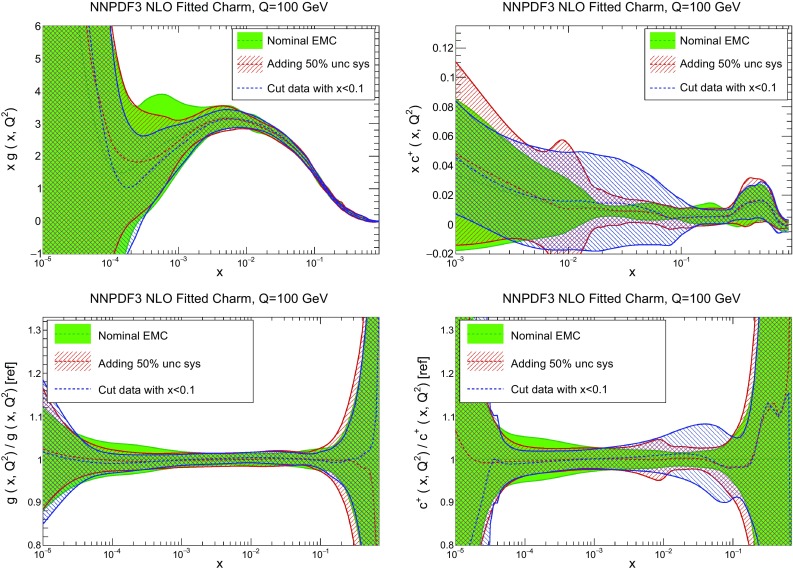
Same as Fig. 3, but now comparing the default results with fitted charm with those obtained removing all EMC data with $x<0.1$, or adding and extra 50% systematics to all EMC data

The original EMC charm structure functions were obtained assuming an inclusive branching fraction of *D* mesons into muons, $\mathrm{BR}(D\rightarrow \mu +X)=8.2\%$, which differs from the current PDG average [90] and the latest direct measurements from LHCb [91, 92] of the fragmentation probabilities and branching fractions of *D* mesons, which give a value of around 10%. To verify the impact of using these updated branching fractions, and estimate also the possible impact of the other effects, we have rescaled the EMC data by a factor 0.82 and added an additional uncorrelated 15% systematic uncertainty due to $\mathrm{BR}(D\rightarrow \mu +X)$. The results are also shown in Fig. 12, where we see that this rescaling has only a small impact on the charm PDF. The impact becomes completely negligible if the systematics is taken to be correlated [89].

Since the charm data were taken on an iron target, nuclear corrections should be applied, as is the case also for the various fixed-target neutrino datasets included in our global fit: in fact, in the smallest *x* bins, shadowing corrections could be as large as 10–20% (see e.g. Ref. [93]). Furthermore, it was argued in Ref. [12] that higher-twist corrections obtained by replacing $m_c^2$ by $m_c^2\left( 1+\frac{\Lambda ^2}{m_c^2}\right)$ (where $\Lambda \sim 200$ MeV is a binding energy scale) may have a substantial effect on the lowest $Q^2$ (and thus smallest *x*) EMC data. Finally, of course, the EMC data have been obtained using analysis techniques which are quite crude to modern standards, for example only relying on LO QCD computations. The latter caveat, however, is in fact common to all the oldest fixed-target deep-inelastic scattering data which are still currently used for PDF determination, such as SLAC [38] and BCDMS [94, 95], for which there is no evidence (see in particular Table 10 of Ref. [1]) that systematics are significantly underestimated, though, of course, specific issues only affecting EMC (such as the aforementioned background estimation) cannot be excluded.

In order to explore possible consequences of missing corrections (such as nuclear or higher twist), or uncertainty underestimation, we have performed two more fits. In the first, we have removed all EMC data with $x<0.1$, namely the region where nuclear and higher-twist corrections are largest. In the second, we have retained all EMC data, but with an extra 50% correlated systematics. Results are shown in Fig. 13. It is clear that the effect of the added systematics is minor: the percentage increase of uncertainties is moderate, and the central value changes very little. On the other hand, as one might expect, removing the small-*x* EMC data leaves the best-fit charm unchanged for $x>0.1$, but for smaller *x* it leads to results which are similar to those (shown in Fig. 12) when the EMC data are not included. This shows that the large *x* EMC data are responsible for the large *x* bump, while the small *x* EMC data are responsible for the small *x* undershoot in comparison to the perturbative charm case.

We conclude that, while we have no direct evidence that uncertainties in the EMC data might be underestimated, and specifically not more than for any other old deep-inelastic scattering dataset, there are persuasive theoretical arguments which suggest that these data might be affected by significant nuclear or higher-twist corrections, especially at small *x*. However, we find that even a very substantial increase of the systematic uncertainty of this data does not change its qualitative impact, as one might perhaps expect given the very large discrepancy between the data and predictions obtained with purely perturbative charm at small and large *x*. On the other hand, until more data are available phenomenological conclusions based on this data should be taken with a grain of salt, as is always the case when only a single dataset is responsible for a particular effect: as seen in Fig. 11, about half a dozen points are mostly responsible for the effect seen at small *x* and as many at large *x*. However, regardless of the actual reliability of these data, there remains an issue of principle: if the EMC results were true, to what extent might the assumption of perturbative charm bias the fit result? This question is addressed in the next subsection.

### The charm PDF and its intrinsic component

**Fig. 14:**
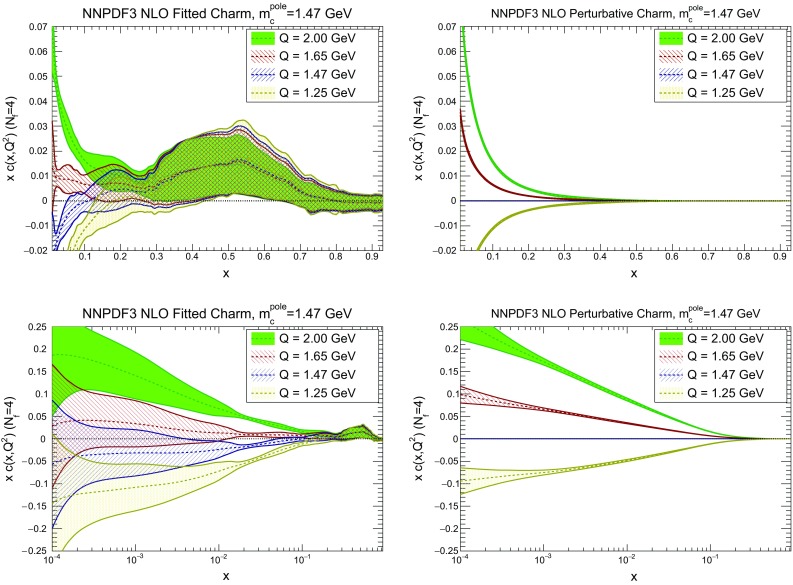
The charm PDF (when $m_c=1.47$ GeV) plotted as a function *x* on a linear (*top*) or logarithmic scale (*bottom*) for four low scale values $Q=1.25$, 1.47, 1.65 and 2 GeV in the four-flavour scheme. Both fitted (*left*) and perturbative (*right*) charm are shown. Note that in a matched scheme the charm PDF would become scale independent for $Q<m_c$

We now discuss the qualitative features of the best-fit charm PDF. Our goal here is not to assess the reliability of the data on which it is based (which was discussed in the previous subsection) but rather to examine the implication of a scenario in which such data are assumed to be true. Such a scenario does not appear to be forbidden or unphysical in any sense, so it is interesting to ask whether in this scenario a PDF determination without fitted charm would lead to biased results.

**Fig. 15:**
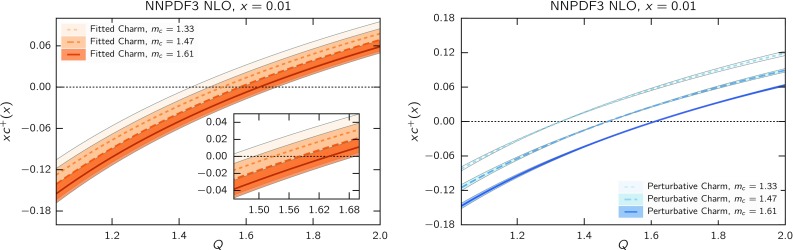
The charm PDF in the four-flavour scheme as a function of scale at $x=0.01$ for different values of the heavy quark mass with fitted (*left*) and perturbative (*right*) charm

In order to get a first qualitative assessment, in Fig. 14 the charm PDF is plotted as a function of *x* for various scales close to the threshold. Results are shown, for illustrative purposes, in the four-flavour scheme: in the three-flavour scheme the PDF would become scale independent. Both the fitted (left) and the perturbative (right) charm PDF are shown. The plot is produced from the fitted PDFs by backward evolution using APFEL from the scale $Q=1.65$ GeV. Recall that the independence of the NNPDF results on the scale at which PDF are parametrized is a feature of the NNPDF approach which has been repeatedly verified; see e.g. Ref. [1].

The plot vs. *x* on a logarithmic scale, in which the small *x* region is emphasized, shows that for all $x\lesssim 10^{-1}$ the fitted charm lies below the perturbative charm. However, a scale $Q_0$ at which fitted charm vanishes for all *x* in this region does appear to exist, but it is rather higher, around $Q_0\sim 1.6$ GeV. Recalling that the dependence of the size of the charm PDF at small *x* on the value of charm mass is very considerably reduced when charm is fitted (see Fig. 6), this is a genuine feature, which follows from the data. Of course, in the case of perturbative charm the scale at which the PDF vanishes is instead determined by the value of the mass, as is clear from the right plots of Fig. 14.

The plot vs. *x* on a linear scale, in which the large *x* region is emphasized, in turn shows that the fitted charm PDF displays an ‘intrinsic’ bump, peaked at $x\sim 0.5$ and very weakly scale dependent. This bump is of course absent when charm is generated perturbatively.

The impact of the EMC data on the features of the charm PDF shown in Fig. 14 can be traced to the behaviour shown in Fig. 11 and discussed in Sect. 3.3. Namely, at medium-*x* and low-$Q^2$ the EMC data undershoot the prediction obtained using perturbative charm, while at large-*x* and large $Q^2$ they overshoot it. This leads to a fitted charm which is significantly larger than the perturbative one at large *x*, but somewhat smaller at low *x*.

**Fig. 16:**
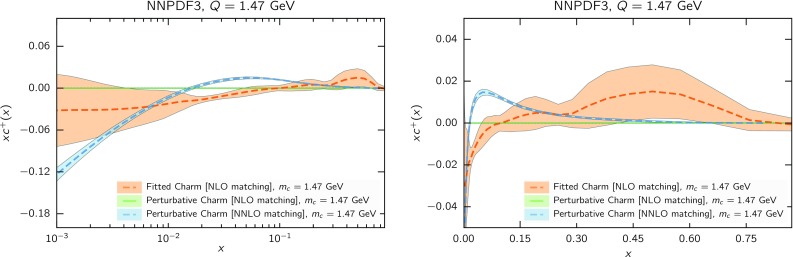
The charm PDF plotted vs. *x* on a logarithmic (*left*) or linear (*right*) scale, when $Q=m_c=1.47$ GeV. The fitted and perturbative NLO and NNLO (see *text*) results are compared

We now discuss each of these features in turn. To elucidate the small *x* behaviour, in Fig. 15 we plot the charm PDF as a function of the scale *Q* for fixed $x=0.01$, for the three values of the charm mass that have been considered above in Sect. 3.2. It is clear that, as mentioned, when charm is fitted (left) the scale at which the PDF vanishes is quite stable, while when charm is perturbative (right) the PDF is very sensitive to the value of the mass since the PDF is constrained to vanish at $Q=m_c$. Specifically the exact scale at which fitted charm vanishes at $x=0.01$ turns out to be $Q_0=1.59$ GeV (when $m_c=1.47$ GeV).

In order to better understand the meaning of this result, in Fig. 16 we compare at the scale $Q=m_c=1.47$ the fitted charm to its perturbative counterpart determined at NLO and NNLO. While the NLO result vanishes by construction, the NNLO result (which will refer to as “NNLO perturbative charm” for short) is obtained using NNLO matching conditions [96, 97] from our best-fit perturbative charm NLO PDF set. Within the FONLL-B accuracy of our calculation, this NNLO charm is subleading, hence it provides an estimate of the expected size of missing higher-order corrections on perturbatively generated charm.

It is interesting to observe that fitted charm for $x\lesssim 0.2$ is similar in size to NNLO perturbative charm, and it has in fact the same (negative) sign for $x\lesssim 0.02$. Of course, to the extent that fitted charm might reabsorb missing higher-order corrections, it would do so not only for matching terms but also for missing corrections to hard matrix elements, which are of the same order and likely of similar size. It is nevertheless intriguing that the observed undershoot of fitted charm when compared to perturbative charm is a feature of the NNLO matching condition at sufficiently small *x*.

All this suggests that our best-fit fitted charm at small *x* is compatible with perturbative behaviour with either a somewhat larger value of the charm mass, or missing higher-order corrections reabsorbed into the initial PDF or a combination of both. This means that if uncertainties related to missing higher orders and the charm mass value were included in perturbative charm, then our fitted charm would be compatible with perturbative charm, but possibly more accurate (in view of the greater stability seen in Fig. 15 of the fitted charm in comparison to the perturbative one). If instead uncertainties related to missing higher orders and the charm mass value are not included (as it is now the case for most PDF sets, including NNPDF3.0) then the charm PDF, within the given uncertainty, is biased (assuming the EMC data are correct).

We now turn to the large *x* behaviour. The fact that our fitted charm has an “intrinsic” component means that it carries a non-negligible fraction of the proton’s momentum. In order to quantify this, we compute the momentum fraction carried by charm, defined as

5 $$\begin{aligned} C(Q^2) \equiv \int _0^1 \mathrm{d}x\, x \left[ c(x,Q^2)+\bar{c}(x,Q^2)\right] . \end{aligned}$$

**Fig. 17:**
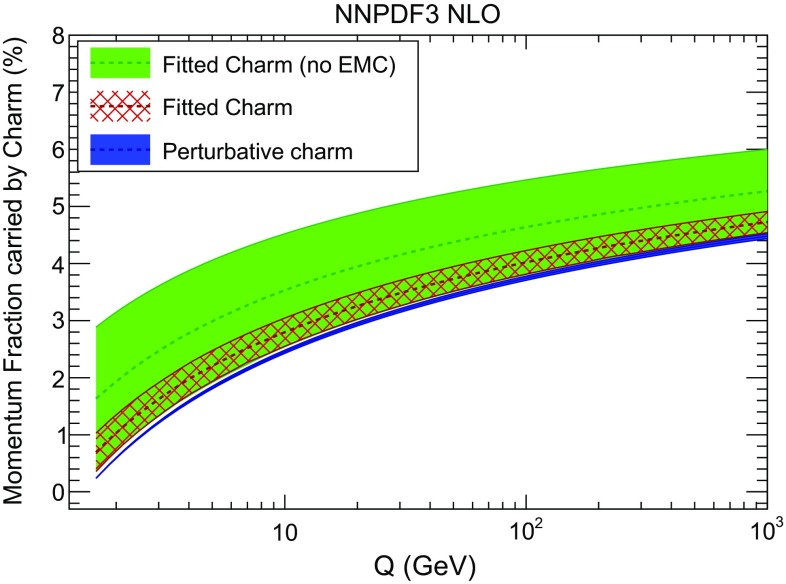
The charm momentum fraction $C(Q^2)$, Eq. (5), as a function of scale with perturbative and with fitted charm, with and without the EMC data included in the fit

**Fig. 18:**
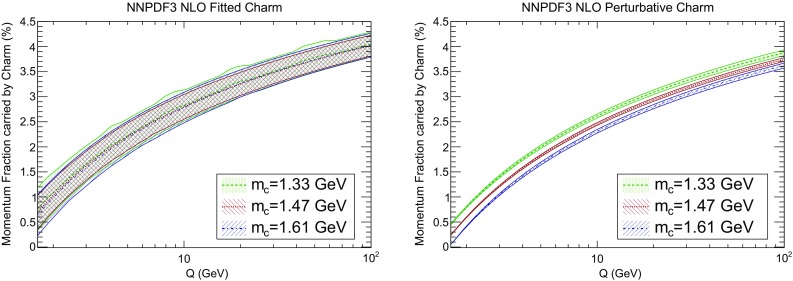
Same as Fig. 17, with three different values of the pole charm mass, for fitted (*left*) and perturbative (*right*) charm

Of course for scales significantly above threshold, both the intrinsic and the perturbative components of the charm PDF will contribute. The momentum fraction $C(Q^2)$ Eq. (5) is plotted as a function of the scale *Q* in Fig. 17, both for fitted and perturbative charm. In the case of fitted charm, results are shown both with and without the EMC data. In Fig. 18 we then show the momentum fraction with the three different values of the charm mass considered in Sect. 3.2.

**Table 3:** The charm momentum fraction $C(Q^2)$ at a low scale $Q=1.65$ GeV with perturbative charm, and with fitted charm with and without the EMC data included. The momentum fractions for several CT14IC PDF sets are also given for comparison (see text)

PDF set	$C(Q=1.65~\mathrm{GeV})$
NNPDF3 perturbative charm	$\left( 0.239 \pm 0.003\right)$%
NNPDF3 fitted charm	$\left( 0.7 \pm 0.3\right)$%
NNPDF3 fitted charm (no EMC)	$\left( 1.6 \pm 1.2\right)$%
CT14IC BHPS1	1.3%
CT14IC BHPS2	2.6%
CT14IC SEA1	1.3%
CT14IC SEA2	2.2%

The values of the momentum fraction at a low scale $Q=1.65$ GeV just above the charm mass, using the central value $m_c=1.47$ GeV are collected in Table 3: they show that both with and without the EMC data we find evidence for intrinsic charm at about the one-sigma level. The intrinsic charm contribution to the momentum fraction, when the EMC data are included, is then around $0.5\pm 0.3\%$, entirely consistent with a power suppression of order $\Lambda ^2/m_c^2$. Without the EMC data, the fraction increases to $1.4 \pm 1.2\%$ (taking into account the perturbative contribution at this scale), though the allowed range for *C*(*Q*) is reduced once the EMC data are included.

At high scale, as shown in Fig. 17, the momentum fraction carried by the charm PDF is dominated by its perturbative component, and it becomes about $5\%$ at $Q=1$ TeV. However, it is clear from Fig. 18 that the momentum fraction of fitted charm is essentially independent of the charm mass at all scales, and is thus determined exclusively by the data. On the other hand, with perturbative charm the momentum fractions obtained for different values of the mass do not overlap at the one-sigma level, even at high scale, and they are thus instead determined by the assumed value of the mass.

In order to further understand the features of our fitted intrinsic component we compare it to previous determinations based on models. To this purpose, we compare our fitted charm with the charm PDFs recently given in Refs. [2, 15] within the framework of the CT14 NNLO PDF determination. In this analysis two different models for intrinsic charm were considered: a BHPS scenario [98] in which charm at $Q_0=1.3$ GeV has a valence-like shape,

6 $$\begin{aligned} c(x,Q_0)=Ax^2\left[ 6x(1+x)\ln x+ (1-x)(1+10x+x^2)\right] , \end{aligned}$$

which peaks around $x\sim 0.25$, and a SEA model in which charm is assumed to have the same shape as the light quark sea:

7 $$\begin{aligned} c(x,Q_0)=A\left[ \bar{d}(x,Q_0)+\bar{u}(x,Q_0)\right] . \end{aligned}$$

In both cases, the only free parameter of the model is the positive-definite normalization *A*, for which two different values, corresponding to two different momentum fractions, are considered (see Table 3).

**Fig. 19:**
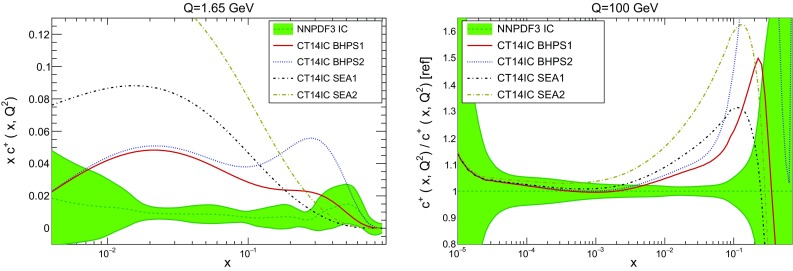
Comparison of the NNPDF3 fitted charm PDF with the different CT14IC models of [2, 15] at a low scale $Q=1.65$ GeV (*left*) and at a high scale $Q=100$ GeV (*right*)

In Fig. 19 we compare the NNPDF3 fitted charm PDF with the four CT14 IC models both at a low scale $Q=1.65$ GeV and at a high scale $Q=100$ GeV. While the fitted charm is qualitatively similar to the BHPS model [98], it is entirely different from the SEA model. At small *x* the NNPDF3 fitted charm is smaller than all the models, and it peaks at larger values of *x* than the BHPS model. At high scale, there is good agreement between our fitted charm and the models in the region where perturbative evolution dominates, $x\lesssim 10^{-3}$, with more substantial differences at medium and large *x*: for example, for $x\simeq 0.2$ the charm PDF in the BHPS1 model is 40% larger than in our fit. Comparing the momentum fractions in Table 3, our fitted charm result with EMC data prefers a rather lower momentum fraction than was considered in Refs. [2, 15]. In fact it seems that the BHPS model, with normalization reduced by 40% or so from that used in BHPS1, might be in reasonable agreement with our fit at large *x*.

We also find that results contradict the claim from the authors of Ref. [14] (based on the JR PDF fit framework) that values of the charm momentum fraction of *C*(*Q*) at the 0.5% level are excluded at the four-sigma level. Note, however, that none of these models reproduce the features of our best-fit charm at small *x*, and specifically the undershoot in comparison to the perturbative behaviour discussed above.

Our general conclusion is thus that if the EMC data are reliable, then charm is compatible with perturbative behaviour at small $x\lesssim 0.1$, where it vanishes at a scale which at NLO turns out to be $Q_0\sim 1.6$ GeV, while it has an intrinsic component at large *x* which carries about a percent of the proton momentum at low scale. Not including a fitted charm component with $m_c=1.47$ GeV would thus bias the PDF determination both at small and large *x*, with the large *x* bias localized at low scale and the small *x* bias also affecting high-scale physics. The small *x* bias would, however, mostly disappear if PDFs were provided with uncertainties related to missing higher-order corrections and the value of the charm mass, or if the mass value was raised.

**Fig. 20:**
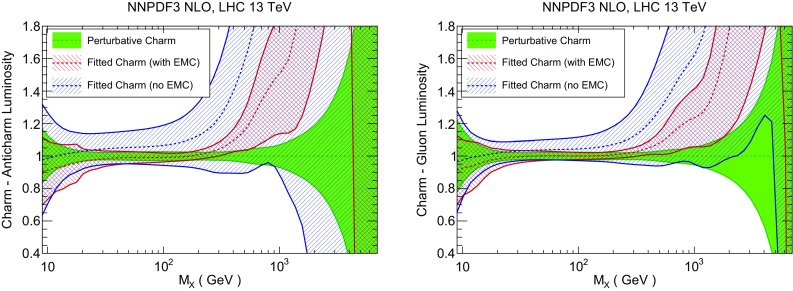
Parton luminosities at the LHC 13 TeV as a function of the invariant mass $M_X$ of the final state, computed using the PDF sets with perturbative charm, and with fitted charm with and without EMC data. The charm–anticharm (*left*) and charm–gluon luminosities (*right*) are shown

**Fig. 21:**
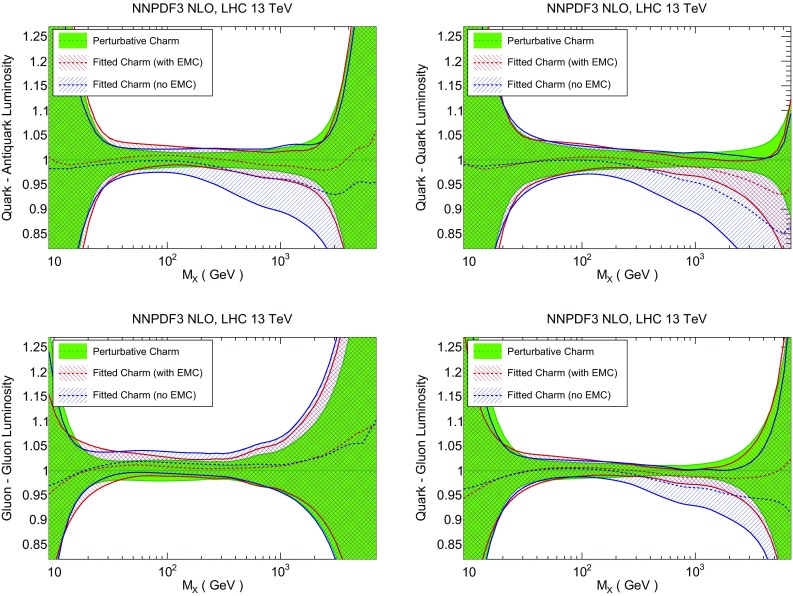
Same as Fig. 20, but for quark–antiquark (*top left*), quark–quark (*top right*), gluon–gluon (*bottom left*) and quark–gluon (*bottom right*) luminosities

## LHC phenomenology

We now discuss the implications of fitting charm for LHC phenomenology. First, we compare parton luminosities computed with fitted or perturbative charm, and specifically show at the level of luminosities the improved stability upon variation of the charm mass that was already discussed in Sect. 3.2 at the level of PDFs. We then turn to specific processes: first, we discuss the effect of fitting charm on standard candles, thereby showing that fitting charm is advantageous for more robust uncertainty estimation. Then we consider representative LHC processes which are sensitive to charm and could be used for a more accurate charm PDF determination: charm quark pair production and *Z* production in association with charm quarks.

**Fig. 22:**
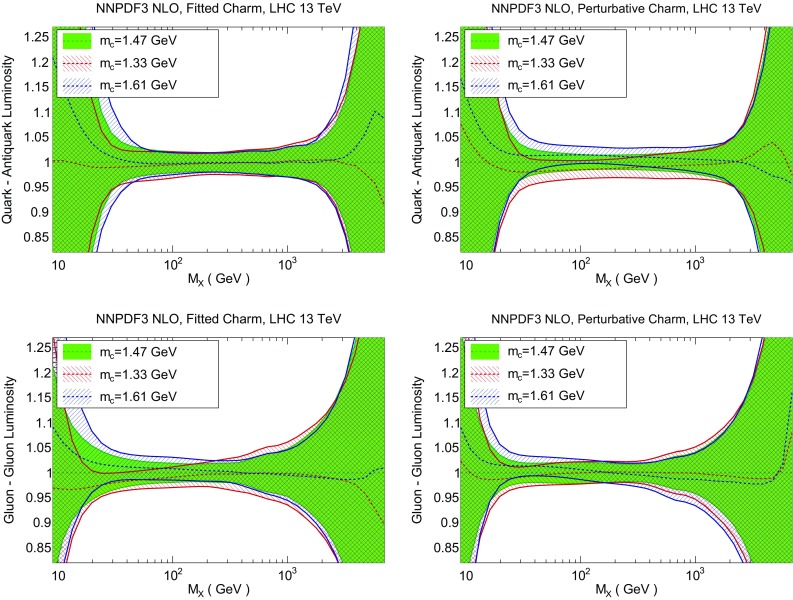
Same as Fig. 21, but now comparing the quark–antiquark (*top*) and gluon–gluon (*bottom*) luminosities for different values of the pole charm mass, for fitted (*left*) and perturbative charm (*right*)

**Fig. 23:**
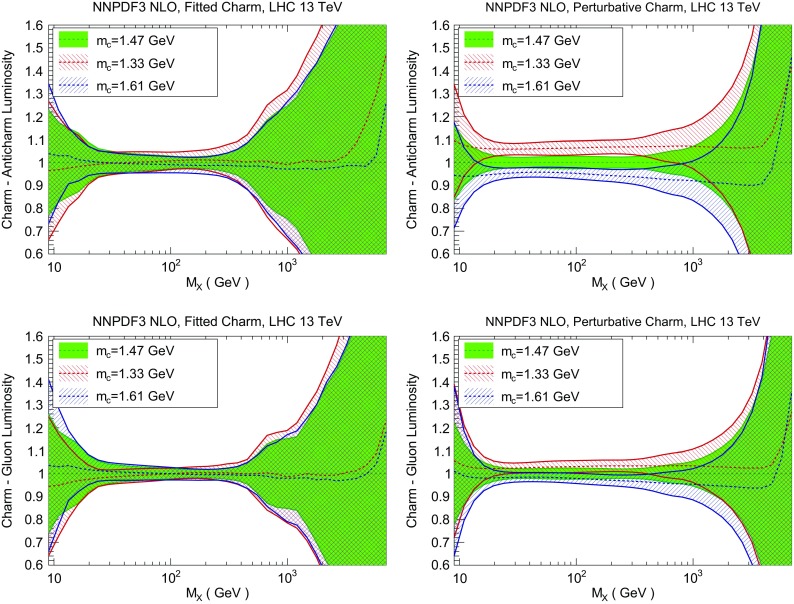
Same as Fig. 22 for charm–anticharm (*top*) and charm–gluon luminosities (*bottom*)

### Parton luminosities

In Fig. 20 we compare parton luminosities (defined as in Ref. [99]) involving charm at the LHC 13 TeV, plotted as a function of the invariant mass $M_X$ of the final state, for the PDF sets with perturbative and fitted charm, with and without EMC data, discussed in Sect. 3. We show the charm–anticharm and charm–gluon luminosities, which are relevant for charm-dominated processes at the LHC, such as *D* meson production at large $p_T$ and rapidity, where the $c\bar{c}$ process becomes important, or $\gamma /Z+D$ production, which at the Born level is driven by the *gc* luminosity. We find that when the EMC data are included, the uncertainty in the luminosity with fitted charm is similar to that when charm is perturbative for scales $M_X\sim 100$ GeV, and larger than it by a factor 3 or 4 for higher or lower scale, while if EMC data are not included the uncertainty with fitted charm is substantially larger for all scales. This suggests that the determination of the luminosities with purely perturbative charm might be unreliable, with underestimated uncertainty and possibly a biased central value, particularly at high invariant masses. In Fig. 21 we show luminosities involving light quarks and gluons. In this case, the uncertainties are similar with fitted or perturbative charm provided EMC data are included.

The difference between fitted and perturbative charm is particularly apparent in the dependence of luminosities on the value of the charm mass, which is shown in Fig. 22 (for the light quark–antiquark and the gluon–gluon luminosity). A marked increase in stability is seen in the $q\bar{q}$ luminosity for all $M_X$ when charm is fitted. This means that if charm is not fitted, the choice of charm mass is a possible source of bias. The reduced dependence on the value of $m_c$ becomes especially striking for luminosities involving charm: as shown in Fig. 23, the spread in central values for the charm–anticharm luminosity as the charm mass is varied is about 15% for perturbative charm and about 2% for fitted charm for all 20 GeV$<M_X<$1 TeV. Similar conclusions hold for the *cg* luminosity.

### LHC standard candles

We now study the impact of the fitted charm PDFs for the calculation of standard candles at the LHC. We start with total cross sections and then consider some differential distributions, all at the LHC 13 TeV.

#### Total cross sections

**Fig. 24:**
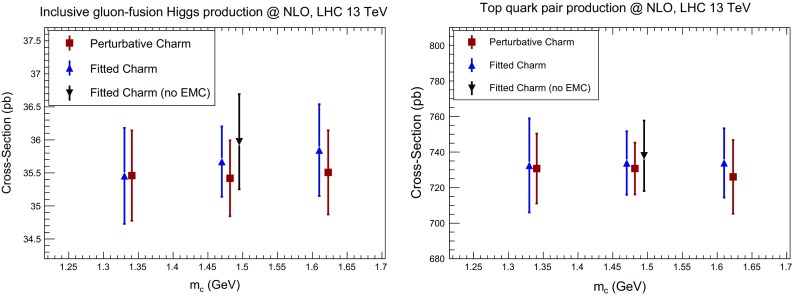
The NLO cross sections for Higgs production in gluon fusion (*left*) and inclusive top quark pair production (*right*) at the LHC 13 TeV with fitted or perturbative charm and $m_c^\mathrm{pole}= 1.33, 1.47$ and 1.61 GeV. We also show the result with fitted charm and no EMC data for $m_c^\mathrm{pole}= 1.47$ GeV. The uncertainty shown is the PDF uncertainty only (not including i.e. $\alpha _s$ variations)

We first consider Higgs and top production. We have computed the total inclusive Higgs production cross section in the gluon fusion channel using the ggHiggs code v3.2 [100] to NLO, including full dependence on the top, bottom and charm masses, for $\mu _F=\mu _R=m_H/2$ and $m_H=125$ GeV. We have also computed the inclusive top quark pair production cross section at NLO using top++ v2.0 [101]. Results are collected in Table 4 and represented in Fig. 24. Note that the uncertainty shown is the PDF uncertainty only (not including $\alpha _s$ variation). In both cases, the impact of fitting charm on the cross section is moderate, both for central values and uncertainties, and while the cross section is almost independent of the charm mass for perturbative charm, it varies a little more when the charm is fitted. The overall uncertainty is thus a little larger with fitted charm, reflecting the slightly increased uncertainty in the gluon–gluon luminosity.

**Table 4:** Numerical values for the cross sections represented in Figs. 24 and 25

Process	Charm PDF	$m_c=1.33$ GeV	$m_c=1.47$ GeV	$m_c=1.61$ GeV
$\sigma (gg\rightarrow h)$ [pb]	Fitted	$35.5\pm 0.7$	$35.7 \pm 0.5$	$35.8 \pm 0.7$
	Fitted (no EMC)	–	$36.0 \pm 0.7$	–
	Perturbative	$35.5 \pm 0.7$	$35.4 \pm 0.6$	$35.5 \pm 0.6$
$\sigma (t\bar{t})$ [pb]	Fitted	$733\pm 26$	$734 \pm 18$	$734 \pm 20$
	Fitted (no EMC)	–	$738 \pm 20$	–
	Perturbative	$731 \pm 20$	$731 \pm 15$	$726\pm 21$
$\sigma (W^+\rightarrow l^+\nu )$ [nb]	Fitted	$6.09\pm 0.14$	$6.14\pm 0.13$	$6.04\pm 0.13$
	Fitted (no EMC)	–	$6.15\pm 0.12$	–
	Perturbative	$5.97\pm 0.10$	$6.03\pm 0.10$	$6.11\pm 0.10$
$\sigma (W^-\rightarrow l^-\nu )$ [nb]	Fitted	$4.42\pm 0.10$	$4.43\pm 0.09$	$4.40\pm 0.09$
	Fitted (no EMC)	–	$4.44\pm 0.08$	–
	Perturbative	$4.38\pm 0.07$	$4.41\pm 0.07$	$4.47\pm 0.07$
$\sigma (Z\rightarrow l^+l^-)$ [nb]	Fitted	$1.412\pm 0.028$	$1.410\pm 0.026$	$1.410\pm 0.025$
	Fitted (no EMC)	–	$1.400\pm 0.023$	–
	Perturbative	$1.376\pm 0.022$	$1.380\pm 0.021$	$1.5403\pm 0.021$

**Fig. 25:**
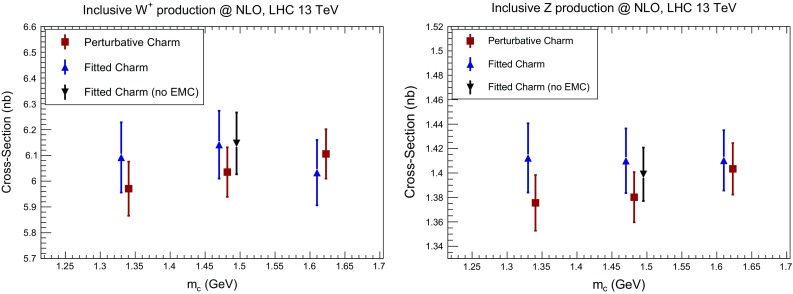
Same as Fig. 24 for the cross section for the inclusive production of $W^+$ (*left*) and *Z* (*right*) bosons at the LHC 13 TeV, including leptonic branching fractions and standard acceptance cuts

Next, we have computed the total cross section for *W* and *Z* production at NLO at the LHC 13 TeV using MCFM [102]. We include the leptonic decays of the gauge bosons, and we impose standard acceptance requirements for the final-state leptons, namely $p_T^l \ge 20$ GeV and $|\eta _l|\le 2.5$.

Results are presented in Fig. 25 and collected in Table 4. Here, while again results with perturbative or fitted charm are very similar, an improvement in stability with respect to the choice of $m_c$ when charm is fitted is clearly visible for *Z* production. Also, we see that whether or not we include the EMC data makes very little difference to these standard candles.

As a general conclusion, we find that the variation of total cross section for LHC standard candles as the charm mass is varied in a very conservative range is a small fraction of the PDF uncertainty. This conclusion is in agreement with previous studies of the dependence of global fit results on the charm mass (but with perturbative charm only) presented in Refs. [80, 103–105].

#### Differential distributions

We now turn to differential distributions for Higgs production in gluon fusion, top-pair production and *W*, *Z* electroweak gauge-boson production at 13 TeV. All calculations have been performed at NLO using MadGraph5_aMC@NLO [106] interfaced to aMCfast [107] and APPLgrid [71]. The choice of binning, kinematical cuts and final-state decays in these processes are the same as those used in the SM-PDF study [108], to which we refer for further information. In each case, we compare results obtained with perturbative charm, and with fitted charm when EMC data are included or not. All uncertainties shown are PDF uncertainties only. In addition, we also compare results for fitted charm (with and without EMC data) obtained with different values of the charm mass, and the corresponding results in the case of fits with perturbative charm.

**Fig. 26:**
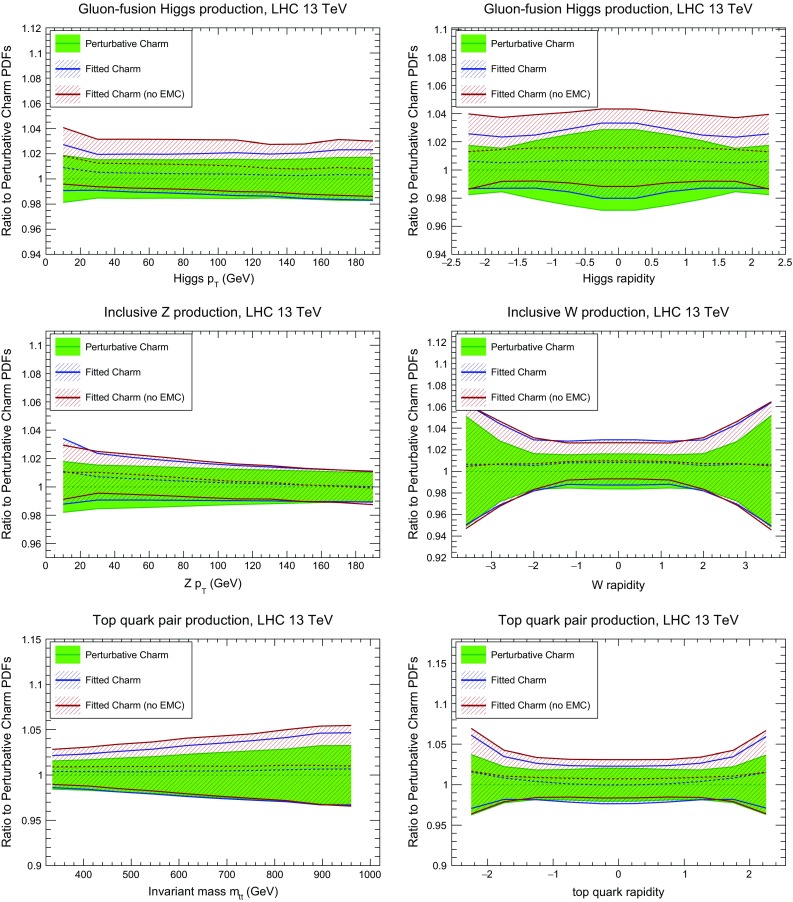
Comparison of the results of the baseline fit with perturbative charm with the corresponding fitted charm PDFs, with and without the EMC data included for NLO differential distributions at 13 TeV. From *top* to *bottom* and from *left* to *right* we show the Higgs transverse momentum and rapidity, the $p_T$ of the *Z* boson, the rapidity of the *W* boson, and $m_{t\bar{t}}$ and $y_t$ in $t\bar{t}$ production

**Fig. 27:**
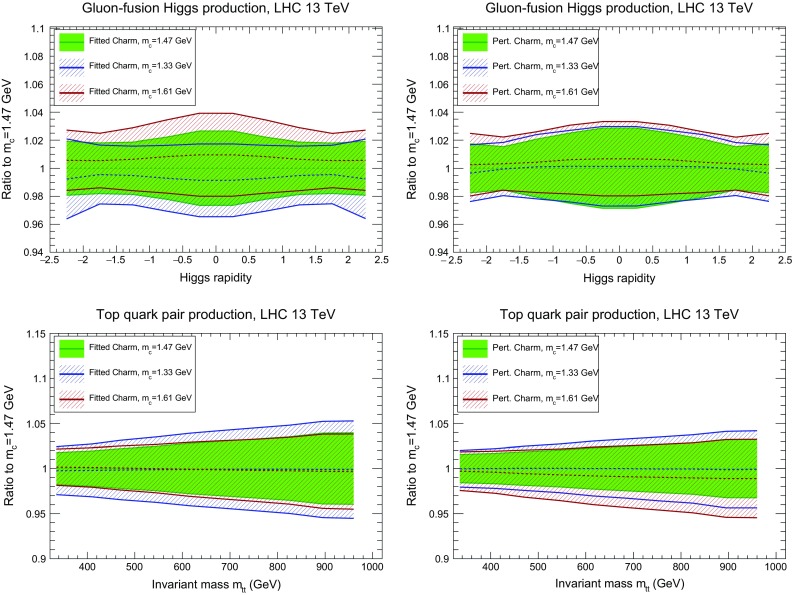
The Higgs rapidity distribution and the invariant mass distribution of top quark pairs in $t\bar{t}$ production, as in Fig. 26, but now comparing different values of the charm mass with fitted charm (*left*) and perturbative charm (*right*)

**Fig. 28:**
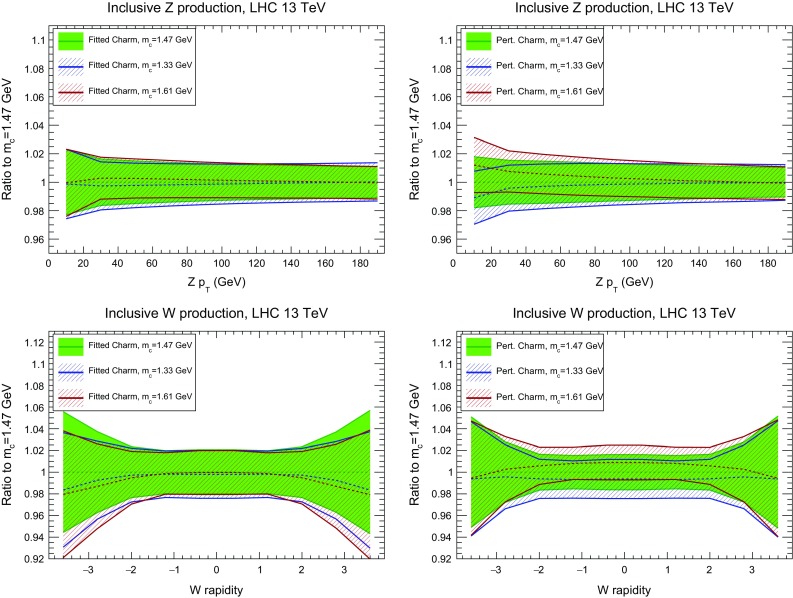
Same as Fig. 27 for the $p_T$ of the *Z* boson and the rapidity of the *W* boson

In Fig. 26 we show the Higgs transverse momentum and rapidity, the transverse momentum of the *Z* boson, the rapidity of the *W* boson, and the invariant mass and top quark rapidity in $t\bar{t}$ production. In all cases, we observe considerable stability of central values when moving from perturbative to fitted charm, with only a small increase in uncertainty for fitted charm, and no significant difference found when EMC data are excluded.

Then in Figs. 27 and 28 we show the comparison of the differential distributions of Fig. 26 upon variations of the charm quark mass, both for fitted and perturbative charm PDFs. For the gluon-initiated processes (*ggH* and $t\bar{t}$) the results with fitted and perturbative charm are quite similar: the main effect of fitted charm is to give a more conservative estimate of the overall uncertainty. For quark-induced processes (*W* and *Z*) we see a marked improvement in the stability upon charm mass variations for fitted charm, particularly at low $p_T$ and at central rapidities: this is a direct reflection of the reduced sensitivity to charm mass variations in the medium *x* region when charm is fitted.

We conclude that for LHC observables which do not depend directly on the charm PDF, both at the inclusive and differential level, the impact of fitting charm is moderate: for gluon dominated processes it provides a more conservative error estimate, while for quark-induced processes it offers a reduction in the (already quite weak) dependence on the value of $m_c$.

### Probing charm at the LHC

We now turn to LHC observables which do depend directly on the charm PDF, and which could thus be used for its determination. Such observables include prompt photon production in association with *D* mesons [109–111], *Z* boson production together with charm quarks [112–114] and open *D* meson production [115–119], as well as more exotic processes such as double charmonium production [21], and inclusive and diffractive Higgs production [120, 121]. Here we concentrate on two illustrative cases, namely *Z*+charm and $c\bar{c}$ production. We will specifically discuss the kinematic regions which are sensitive to the charm PDF at large *x*, and which could therefore be used to confirm our first evidence, discussed in Sect. 3.4, for an ‘intrinsic’ component of charm: as we will see, these are the regions of large $p_T$ or large rapidity.

#### *Z* production in association with charm quarks

**Fig. 29:**
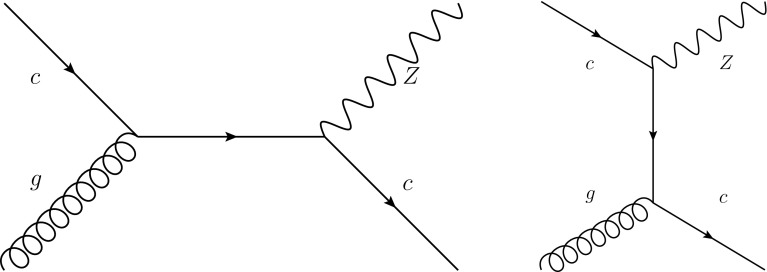
Representative leading-order Feynman diagrams for the production of a *Z* boson in association with a charm quark at hadron colliders

**Fig. 30:**
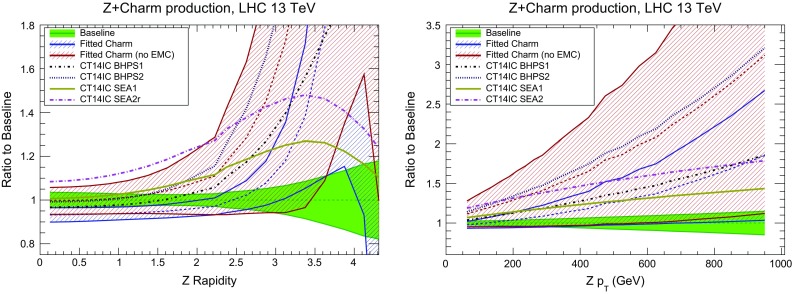
The *Z* boson rapidity (*left*) and transverse momentum (*right*) distributions for *Z* production in association with charm at the LHC 13 TeV, computed using the NNPDF sets with perturbative or fitted charm, and the CT14 IC PDFs shown in Fig. 19. Results are shown as a ratio to the NNPDF perturbative charm set

In Fig. 29 we show representative leading-order Feynman diagrams for the production of a *Z* boson in association with a charm quark at hadron colliders, driven by the *cg* luminosity. The calculation of this process at NLO has been performed with MCFM interfaced to APPLgrid, and cross-checked with MadGraph5_aMC@NLO interfaced to aMCfast. It is beyond the scope of this paper to perform a complete feasibility study of this measurement, so we neglect the hadronization of the charm quark into a *D* meson, which does not significantly affect the sensitivity of this process to the charm PDF. In Fig. 30 we show the rapidity distribution and the transverse momentum of the *Z* boson in $Z+c$ production at the LHC 13 TeV. We compare the results of perturbative or fitted charm PDFs, in the latter cases with and without the EMC data included. We also show predictions obtained using the four CT14NNLO sets discussed in Sect. 3.4.

In the case of the *Z* rapidity distribution percentage differences in central values are moderate at central rapidity but increase substantially in the forward region. In particular, in the LHCb acceptance region, $2.0 \le y_Z \le 4.5$, an enhancement of the cross section by a factor 2 or more is possible in the case of fitted charm, compared to the baseline result with perturbative charm (for a recent study of charm PDF constraints in $Z+c$ production at LHCb see e.g. Ref. [112]). However in this region PDF uncertainties in the fitted charm case are large, and the three NNPDF sets shown agree with each other at the one-sigma level in the entire range of $y_Z$. This means that more accurate data for this observable could provide a useful constraint on the charm PDF.

In the case of the transverse momentum distribution of *Z* bosons, the NNPDF sets with fitted charm and the CT14 sets based on the BPHS model exhibit a substantial enhancement of the cross section at large $p_T^Z$ in comparison to the perturbative charm baseline. For the fitted charm NNPDF3 PDFs with EMC data, this enhancement could be as large as a factor 2 (at the one-sigma level) for $p_T^Z\simeq 700$ GeV. Once again, however, results obtained with perturbative and fitted charm PDFs are consistent with each other within the large uncertainties, so also in this case more accurate measurements could provide a useful constraint.

Turning things around, an accurate measurement at high rapidity and transverse momentum could rule out perturbative charm. Also, in the central rapidity region, an accurate enough measurement could confirm the undershoot in the fitted charm case which is seen in Fig. 30, and, though smaller in absolute terms, it is as significant as the large rapidity excess on the scale of present-day uncertainties. A full NNLO analysis will be required in order to arrive at a definite conclusion, especially in view of the fact that, as discussed in Sect. 3.4, the fitted charm might be reabsorbing higher-order corrections.

#### Charm quark pair production

**Fig. 31:**
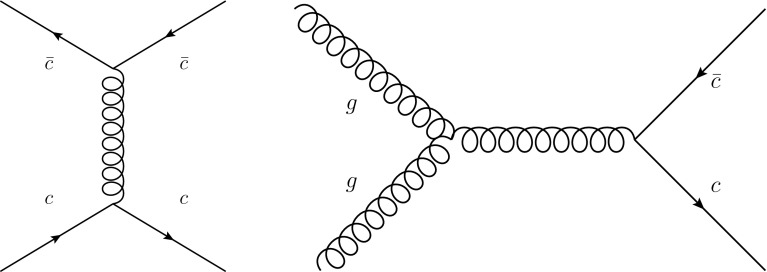
Representative leading-order Feynman diagrams for the production of a charm–anticharm pair at hadron colliders, initiated either by charm quarks (*left*) and by gluons (*right*)

At hadron colliders, heavy quark pair production is driven by the *gg* and $q\bar{q}$ luminosities. The relative importance of the two channels depends on the kinematics. For instance, for the total inclusive cross section in top quark pair production [122], the *gg* process is dominant at the LHC 13 TeV (90%), while it is only 14% at the Tevatron (where instead 86% of the cross section comes from quark-initiated contributions). In the case of charm quark pair production, at low transverse momentum $p_T^c$, the cross section is entirely dominated by gluon-initiated processes [91]. However, in the case of fitted charm the $c\bar{c}$ channel can eventually become dominant for high enough transverse momentum of the charm quark $p_T^c$, or for high enough rapidity $y_c$: in these cases, large values of *x* are probed, where the fall-off of the charm PDF is less steep than that of the gluon, especially if charm has an intrinsic component. Representative leading-order Feynman diagrams for the production of a charm–anticharm pair at hadron colliders are shown in Fig. 31.

In the following, we use the FONLL code [18] for the calculation of the double-differential cross section $\mathrm{d}^2\sigma _{c\bar{c}}/\mathrm{d}p_T\mathrm{d}y$ for the production of a charm–anticharm pair at hadron colliders. The FONLL calculation combines a fixed-order massive result, accurate at small $p_T$, with a resummed next-to-leading log prediction in which the charm mass is neglected. As in the case of deep-inelastic scattering, the massive fixed-order calculation should be modified in the presence of a fitted charm component [22, 23]. This modification is not included in the code of Ref. [18]; here, however, we will only consider the large $p_T\gg m_c$ region, where the FONLL computation coincides with the massless one and this extra contribution is negligible. Since our aim is only to illustrate how differences in the charm PDF affect the charm pair production cross section, we do not include final-state effects such as hadronization of charm quarks into *D* mesons and their subsequent decay.

**Fig. 32:**
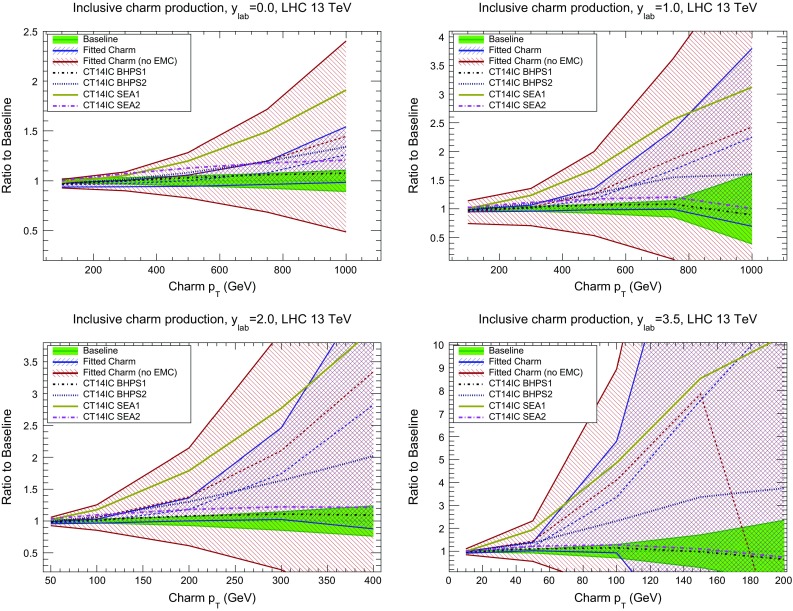
The double-differential cross section $\mathrm{d}^2\sigma _{c\bar{c}}/\mathrm{d}p_T\mathrm{d}y$ for charm–anticharm pair production at the LHC 13 TeV, as a function of the charm quark $p_T$ for different values of its rapidity *y*. From top to bottom and from left to right, we show the results for $y_c=0,1.0,2.0$ and 3.5. We compare results obtained using the NNPDF sets with perturbative or fitted charm, and the CT14 IC PDFs shown in Fig. 19. Results are shown as a ratio to the NNPDF perturbative charm set

In Fig. 32 we show the double-differential cross section $d^2\sigma _{c\bar{c}}/dp_Tdy$ for charm–anticharm pair production at the LHC 13 TeV, as a function of the charm quark transverse momentum for different values of its rapidity $y_c$. The impact of different charm PDFs becomes more important at large $p_T^c$ and for large $y_c$. For instance, for $y_c=3.5$, intrinsic charm can enhance the cross section for charm production by up to one order of magnitude for $p_T^c=200$ GeV.

While *D* meson production in the forward region has been measured by LHCb, available data only cover the kinematic region up to $p_T^D=8$ GeV at 7 TeV [92] and up 15 GeV at 13 TeV [123], where the differences between the fitted and perturbative charm predictions are small. Future LHCb *D* meson production data with higher integrated luminosity and a higher reach in $p_T^D$ could be used to constrain the charm content of the proton. Similarly, *D* meson production in the central region $|\eta _D|\lesssim 2$, but higher $p_T^D$ values than currently available could provide valuable constraints. Note that likewise current ATLAS *D* meson measurements at 7 TeV [124] extend only up to $p_T^D=100$ GeV, so data at 13 TeV with increased luminosity would also be required here.

## Delivery and outlook

We have presented a first model-independent determination of the charm content of the proton in the NNPDF framework. Our results suggest that, if the EMC data are taken at face value, the charm PDF is compatible with perturbative behaviour for $x\lesssim 0.1$, in that it vanishes for all *x* in this region around $Q_0\approx 1.6$ GeV, while it has an ‘intrinsic’ large *x* component which peaks for $x\sim 0.5$, and carries $0.7\pm 0.3$% of the nucleon momentum at the 68% CL at a low scale $Q=1.65$ GeV. The perturbative component of our fitted charm is quite stable upon variation of the charm mass, and thus lies significantly below perturbatively generated charm if the central PDF value $m_c=1.47$ GeV is adopted. This could possibly be due to missing higher-order corrections, which are expected to be of comparable size. This suggests that PDF sets (including NNPDF3.0), in which charm is perturbatively generated but no theoretical uncertainties are provided, may be significantly underestimating the uncertainty on the charm PDF at small *x*, and missing its intrinsic component at large *x*. These results hold even if the uncertainty on the EMC charm data is considerably inflated, and in fact at the level of central values they still hold even with the EMC data excluded altogether, though in that case they lose statistical significance.

Perhaps more interestingly, our results show that the widely held opinion (see e.g. Ref. [14] and Refs. therein) that the EMC data cannot be included in a global fit because they are in tension with other datasets, i.e. they cannot be adequately fit at leading-twist taking both data and theory at face value, is untenable. Indeed, we show that if we take the published EMC $F_2^c$ data and simply include them in an NLO fit based on the FONLL-B scheme with a fitted charm PDF we can fit them perfectly, with a $\chi ^2$ per data point equal to $\chi ^2/N_\mathrm{dat}=1.09$. In other words, regardless of their reliability, the EMC data provide us with an interesting test-case scenario which demonstrates that the perturbative treatment of charm in current PDF fits may fail to satisfy the accuracy standards that are required in order to match the high precision that current PDF uncertainties suggest.

When charm is fitted on the same footing as the other light PDFs, we find a small but non-negligible general improvement in global fit quality, and a very significant improvement in the description of large *x* charm structure function data from EMC, which cannot be fitted otherwise. The dependence of the charm PDF on the value of the charm mass is significantly reduced, and there is also a more modest reduction in the charm mass dependence of light quark PDFs. We also find that, while with fitted charm overall uncertainties on gluon-induced LHC cross sections are a little more conservative, the charm mass dependence of quark-induced processes can be reduced at central rapidity and low $p_T$. This suggests that the fitted charm PDFs will lead to more reliable phenomenology at the LHC, eliminating a possible source of bias from assumptions as regards the origin of charm and the value of the charm mass.

An immediate consequence of our results is that existing determinations of the charm quark mass from deep-inelastic structure functions [24, 73, 104, 125–127] might be affected by underestimated theory uncertainties due to the assumption that charm is generated perturbatively. With this motivation, we plan to perform in the near future a direct determination of the charm mass in the global NNPDF analysis both with fitted and with perturbative charm, using the same approach as for the determination of the strong coupling constant [84, 85].

Inclusion of a fitted charm PDF is planned for future general-purpose global PDF sets from the NNPDF Collaboration. Further measurements which might constrain the fitted charm, in particular $Z+c$ and $c\bar{c}$ production at high $p_T$ and high rapidity, are expected at LHC Run 2. We expect the accuracy of the charm determination to improve substantially in the near future, and the issue of the reliability of the EMC data to be finally settled by these measurements.

The NLO PDFs presented here are available in the LHAPDF6 format [128] from the NNPDF HepForge webpage:

https://nnpdf.hepforge.org/html/nnpdf3ic/nnpdf3ic.html

In particular, we make available the following PDF sets:

- PDF sets with fitted charm, for three different values of the pole charm mass: NNPDF3_IC_nlo_as_0118_mcpole_1330 NNPDF3_IC_nlo_as_0118_mcpole_1470 NNPDF3_IC_nlo_as_0118_mcpole_1610
- PDF sets with identical theory settings as those above, with the only differences being that the charm PDF is perturbatively generated and that the EMC data are excluded, for the same three values of the charm mass: NNPDF3_nIC_nlo_as_0118_mcpole_1330 NNPDF3_nIC_nlo_as_0118_mcpole_1470 NNPDF3_nIC_nlo_as_0118_mcpole_1610
- A PDF set with fitted charm and the central value of the charm quark pole mass $m_c^\mathrm{pole}=1.47$ GeV without the EMC charm data included: NNPDF3_IC_nlo_as_0118_mcpole_1470_noEMC
- PDF sets with fitted charm, for three different values of the running $\overline{\mathrm{MS}}$ charm mass: NNPDF3_IC_nlo_as_0118_mc_1150 NNPDF3_IC_nlo_as_0118_mc_1275 NNPDF3_IC_nlo_as_0118_mc_1400
- PDF sets with identical theory settings as those above, with the only differences being that the charm PDF is perturbatively generated and that the EMC data are excluded, for the same three values of the charm mass: NNPDF3_nIC_nlo_as_0118_mc_1150 NNPDF3_nIC_nlo_as_0118_mc_1275 NNPDF3_nIC_nlo_as_0118_mc_1400
- A PDF set with fitted charm and central theory settings but without the EMC charm data included: NNPDF3_IC_nlo_as_0118_mc_1275_noEMC

These PDF sets are not meant to be used for general-purpose applications, for which the NNPDF3.0 PDF sets are still recommended, but rather for studies related to the charm content of the proton. Specifically, fitted charm PDFs should always be compared with the corresponding baseline fits presented in this publication, in order to have a consistent comparison of two PDF sets with identical theory and methodological settings and only differing in the treatment of charm and the inclusion or not of the EMC charm data.
